# Herd-level seroprevalence of *Fasciola hepatica* and *Ostertagia ostertagi* infection in dairy cattle population in the central and northeastern Poland

**DOI:** 10.1186/s12917-018-1455-7

**Published:** 2018-04-17

**Authors:** Sławomir J. Kowalczyk, Michał Czopowicz, Corinna N. Weber, Elisabeth Müller, Tomasz Nalbert, Andrzej Bereznowski, Jarosław Kaba

**Affiliations:** 1 Laboratory of Veterinary Epidemiology and Economics, Faculty of Veterinary Medicine, Warsaw University of Life Sciences, Nowoursynowska 159c, 02-776 Warsaw, Poland; 2 Laboklin GmbH & Co. KG, Steubenstrasse 4, 97688 Bad Kissingen, Germany

**Keywords:** Bulk-tank milk, ELISA, Grazing, Hay, Haylage

## Abstract

**Background:**

*Fasciola hepatica* and *Ostertagia ostertagi* infections are widespread in cattle population of Europe, however data on their prevalence in Poland are only fragmentary. Therefore, the cross-sectional study was carried out to determine the herd-level seroprevalence of *F. hepatica* and *O. ostertagi* infection in dairy cattle population in the central and north-eastern provinces Poland, and to identify basic local risk factors for these infections. In total, 598 herds were enrolled, 394 (65.9%) in the north-eastern province and 204 (34.1%) in the central province. In each herd the questionnaire survey was conducted and bulk-tank milk (BTM) sample was collected and screened using two indirect immunoenzymatic tests. Optical density ratio (ODR) was regarded as the quantitative proxy of exposure to either of the two parasites.

**Results:**

Both *Fasciola* and *Ostertagia* ELISA ODR in the north-eastern province was significantly higher than ODR in the central province. At the cut-off value of ODR = 0.27 the true herd-level seroprevalence of *F. hepatica* was 79.6% (95% CI: 74.0%, 84.3%) in the north-eastern province and 13.0% (95% CI: 5.3%, 21.7%) in the central province. At the cut-off of ODR = 0.50151 of 188 herds (80.3%, 95% CI: 74.1%, 85.4%) were seropositive for *O. ostertagi* in the north-eastern province and only 70 of 136 herds (51.5%, 95% CI: 43.1%, 59.7%) were seropositive in the central province. Location of a herd in the north-eastern province, longer grazing period practiced in a herd and > 50%-share of grazing grass in roughage were all positively related to the increase in exposure to both parasites. Moreover, the use of hay or haylage as main roughage proved to be positively related to the increase in exposure to *F. hepatica.*

**Conclusions:**

*F. hepatica* and *O. ostertagi* are widespread in cattle population in Poland, however their occurrence at a herd-level varies between different regions of Poland. This diversity can only partly be explained by different herd management, and appears linked to environmental and climate conditions typical for these regions.

**Electronic supplementary material:**

The online version of this article (10.1186/s12917-018-1455-7) contains supplementary material, which is available to authorized users.

## Background

Parasitic diseases have great impact on cattle productivity and welfare in all regions of the world [1]. Infections with two groups of internal parasites – liver flukes and gastrointestinal nematodes – are commonly regarded as most detrimental for dairy cattle [2, 3]. The former group is represented in Europe by an ubiquitous fluke *Fasciola hepatica*, responsible for fascioliasis – a disease usually following a chronic course in cattle and known to reduce both the milk yield and quality [4]. Gastrointestinal nematodes are a very diverse group of roundworms of which *Ostertagia ostertagi*, responsible for pathologic lesions in the abomasum, appears to be of main importance in the temperate climate zone [5, 6].

Both *F. hepatica* and *O. ostertagi* infections are widespread in cattle population of Europe. Herd-level prevalence of *F. hepatica* infection ranges from below 10% in Sweden to almost 90% in Wales [3, 7, 8]. Intensity of exposure to *O. ostertagi* expressed as a quantitative result of bulk tank milk ELISA shows similar spatial trends [9–11] with over 90% of affected herds in Great Britain [12]. Similar risk factors have so far been identified for both infections with access to pastures, grazing season length and climate conditions, especially frequent rainfalls, being the most commonly mentioned [9, 11, 13–17].

Latest spatial analysis indicated Poland as the region with low-to-moderate risk of exposure to *F. hepatica* with the north-eastern part of the country as potentially the high-risk area [14]. Observations from the second half of the twentieth century appear to corroborate this result [18], however, recent large-scale studies allowing to verify this model are lacking. The snail *Galba truncatula*, an intermediate host of *F. hepatica*, is widespread in Poland and appears to be quite often a source of cercariae [19]. *F. hepatica* infection has been diagnosed at individual-animal level in 4–20% cows in north-eastern regions using fecal egg count [20], in 5–65% cows in the south-eastern province and 28% in the southern province of Poland on the basis of post-slaughter examinations [21–24] and in 20–50% cows in central and eastern regions using PCR for fecal samples [25]. However, no herd-level disease survey based on bulk-tank milk testing has so far been published. On the other hand, two studies carried out in cattle herds in the southern Poland [26, 27] showed presence of *O. ostertagi* antibodies in bulk-tank milk of all herds enrolled, with 30–56% herds considered as economically affected by the infection. No data on *O. ostertagi* prevalence from other parts of Poland are available.

Therefore, we decided to carry out the cross-sectional study to determine the herd-level seroprevalence of *F. hepatica* and *O. ostertagi* infection in dairy cattle population in the central and north-eastern Poland, and to identify basic local risk factors for these infections.

## Methods

### Herds and questionnaires

The study was carried out in summer 2012 and 2013 in 2 of 16 Polish provinces – Podlaskie province located in the north-eastern part of the country and Łódzkie province situated in the central Poland. Herds were selected from the lists of farmers who used to sell milk to five large dairies located in the north-eastern (3 dairies) and the central province (2 dairies) and they were enrolled in the study provided that they counted at least 10 adult cows (> 24 month-old) and their owners granted informed verbal consent for participation in the study. Given that the estimated parameter in this study was the quantitative result of an immunoenzymatic test (optical density ratio, ODR), only the lower limit of the sample size required (n) was determined at the 95% level of confidence according to the following formula: *n* = (1.96 × σ)^2^ / M^2^, where σ^2^ stood for the variance in the population and M was the desired size of the 95% margin of error (precision). The minimum required sample size was determined so that the desired precision of the estimation (M) was not lower than 20% of the dispersion of observed ODRs (σ^2^) and equaled 97 herds.

Each herd was visited once in 2012 or 2013. During the visit bulk-tank milk (BTM) samples were collected and the questionnaire survey was conducted personally by the author (SJK). The questionnaire had been developed specifically for this study (Additional file 1) and it included following information: herd size (only adult dairy cows), average milk yield per lactation, grazing policy (recorded as no grazing, grazing roughly half a day, roughly whole day or whole 24 h), length of grazing period (in months), main roughage used (corn silage, haylage or hay), proportion of grazing grass in diet (no grazing grass, < 50% of all roughage, > 50% of all roughage), and treatment against worms used in the herd. The study complied with the Directive 2010/63/EU and the Act of Polish Parliament of 15 January 2015 on protection of animals used for scientific purposes. According to Polish legal regulations no formal approval of the ethical commission regarding participation of cattle herd owners in the study (completing questionnaires) was required since questionnaires applied to animal not human health (verbal information from the National Ethical Commission).

### Bulk-tank milk sampling and serological tests

Bulk-tank milk (BTM) samples were collected into 50 ml Falcon with 1 sodium azide tablet per falcon and centrifuged for 15 min. at 1200×g in a cooling centrifuge set to 4 °C. Then, the fat fraction was carefully moved to the side with the plastic spatula, and the whey fraction was collected with a plastic Pasteur pipette, aliquoted into 2 ml Eppendorf tubes and stored at − 20 °C until testing.

Then, BTM samples were screened using two semi-quantitative indirect immunoenzymatic tests from Boehringer Ingelheim Svanova, Sweden: SVANOVIR®F.hepatica-Ab (all samples), based on excretory and secretory antigens (E/S ELISA), and SVANOVIR®O.ostertagi-Ab (only samples collected in 2013). ELISAs were performed according to manufacturer’s manuals. The results were expressed as optical density (OD) ratio (ODR) calculated with the following formula:

*ODR* = (*OD*_*BTM sample*_ − *OD*_*negative control*_)/(*OD*_*positive control*_ − *OD*_*negative control*_)

ODR was regarded as the quantitative proxy of exposure to either of the two parasites.

To report herd-level seroprevalences for the infections some cut-offs must have been assumed. For *F. hepatica* we set the cut-off value at ODR = 0.27 as for this figure sensitivity and specificity for identifying herds in which more than 25% of the cows were infected was known and equaled 96% (95 CI: 89%, 100%) and 80% (95 CI: 66%, 94%), respectively [8]. Within-herd prevalence of *F. hepatica* of at least 25% had been in turn previously proposed as a threshold for significant production losses [28]. Furthermore, we used the cut-off values recommended by the manufacturer to identify herds affected by *F. hepatica* at low (economically insignificant) or high (economically significant) level, which equaled ODR = 0.30 and ODR = 0.60, respectively.

For *O. ostertagi* we assumed the cut-off recommended by the manufacturer, which equaled ODR = 0.50, and allowed to identify herds economically affected by the parasite.

Nevertheless, all risk analyses were performed using the crude ODRs instead of categorical results based on the aforementioned cut-off values, in order to retain as much information as possible.

### Statistical analysis

Numerical variables were presented as the median, interquartile range (IQR) and range, categorical variables as the count and percentage in the group. Ninety five per cent confidence intervals (95% CI) for the apparent and true prevalence were determined using the Wilson’s score method. Mean ODRs (standard deviation, SD) were also reported to allow comparisons with other studies, however all statistical analyses were non-parametric as ODRs were non-normally distributed according to the Shapiro-Wilk test (*p* < 0.001 for ODRs of both ELISAs except for ODR of *Ostertagia* ELISA in the central province where *p* = 0.201). In the univariable analysis ODR was correlated with numerical variables using the Spearman’s rank-ordered correlation coefficient (r_s_), and compared between two groups of a categorical variable using the Mann-Whitney U test, or between more than 2 groups of a categorical variable using the Kruskal-Wallis test with Dunn’s post-hoc test. Variables identified as linked with ODR at *p*-value below 0.1 in the univariable analysis were offered to the multivariable linear regression analysis on the basis of the backward stepwise procedure. As correlations between explanatory variables were observed, only the one showing stronger link with ODR was entered into the multivariable analysis. Proportion of ODR variation explained by the model was determined by the coefficient of determination adjusted by the number of explanatory variables included in the model (adjusted-R^2^). All statistical tests were two-sided. A significance level (α) was set at 0.05. Statistical analyses were performed in Statistica 12 (StatSoft Inc.) and the figure in the Microsoft Office Excel.

## Results

In total, 598 herds were visited, 274 (45.8%) in 2012 and 324 (54.2%) in 2013. Of them 394 (65.9%) herds were located in the north-eastern province and 204 (34.1%) in the central province. Herd size ranged from 10 to 109 adult dairy cows and herds were larger in the north-eastern (median of 24, IQR from 17 to 32 heads) than in the central province (median of 18, IQR from 14 to 25 heads) (*p* < 0.001). Of herds tested in 2013 188 (58.0%) were located in the north-eastern province and 136 (42.0%) in the central province. Detailed characteristics of herds from both provinces are given in Table 1 and Additional file 2. In any herd neither regular deworming of adult cows was practiced nor any medicine against parasites had been given for at least 3 months preceding sample collection.

**Table 1:** Characteristics of herds included in the study given as the median, interquartile range and range or as the count and percentage of the entire group

	Entire study population (*n* = 598)	Province		*p*
		north-eastern (Podlaskie) (*n* = 394)	central (Łódzkie) (*n* = 204)	
Herd size	21, 15–30 (10–109)	24, 17–32 (10–109)	18, 14–25 (10–100)	< 0.001
Average milk yield	5500, 460–6400 (1700–10,200)	5500, 4500–6400 (1700–10,200)	5200, 4800–6150 (3000–9360)	0.982
Grazing policy				< 0.001
No grazing	264 (44.2%)	118 (30.0%)	146 (71.6%)	
6 h a day	66 (11.0%)	46 (11.7%)	20 (9.8%)	
12 h a day	179 (29.9%)	156 (39.6%)	23 (11.3%)	
24 h a day	89 (14.9%)	74 (18.7%)	15 (7.3%)	
Length of grazing period in months (only grazed herds)	5 (1–8)	5 (1–8)	5 (1–7)	0.023
Main roughage				< 0.001
Haylage	311 (52.0%)	273 (69.3%)	38 (18.6%)	
Corn silage	263 (44.0%)	103 (26.1%)	160 (78.4%)	
Hay	24 (4.0%)	18 (4.6%)	6 (3.0%)	
Proportion of grazing grass in diet				< 0.001
No grazing grass	203 (33.9%)	90 (22.8%)	113 (55.4%)	
Grazing grass < 50% of all roughage	204 (34.1%)	135 (34.3%)	69 (33.1%)	
Grazing grass > 50% of all roughage	191 (32.0%)	169 (42.9%)	22 (11.5%)	

*Fasciola* ELISA ODR in the north-eastern province ranged from 0.062 to 1.516, with the median of 0.596 and IQR from 0.332 to 0.764, and was significantly higher than ODR in the central province which ranged from 0.002 to 0.989, with the median of 0.146 and IQR from 0.098 to 0.334 (*p* < 0.001). Mean ODRs (SD) were 0.553 (0.266) and 0.235 (0.206), respectively.

**Fig. 1:**
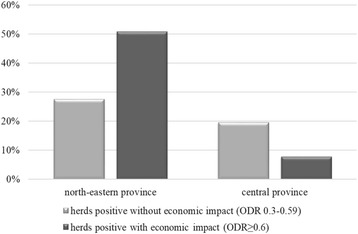
Percentage of herds affected by *Fasciola hepatica* infection in two provinces of Poland. ODR stands for optical density ratio of the ELISA used for screening bulk-tank milk samples

At the cut-off of ODR = 0.27378 herds (63.2%) tested positive for *F. hepatica*, however there was a substantial discrepancy between provinces: as many as 317 of 394 herds (80.5%) were positive in the north-eastern province and only 61 of 204 (29.9%) were positive in the central province. The overall (common for both provinces) true herd-level seroprevalence was 56.9% (95% CI: 52.9%, 60.8%), but it was 79.6% (95% CI: 74.0%, 84.3%) in the north-eastern province and 13.0% (95% CI: 5.3%, 21.7%) in the central province. Also much higher percentage of herds were economically affected by *F. hepatica* infection according to the manufacturer’s cut-offs in the north-eastern province (Fig. 1).

*Ostertagia* ELISA ODR in the north-eastern province ranged from 0.010 to 1.080, with the median of 0.725 and IQR from 0.570 to 0.845, and was significantly higher than ODR in the central province, which ranged from 0.010 to 1.040, with the median of 0.505 and IQR from 0.335 to 0.710 (*p* < 0.001). Mean ODRs (SD) were 0.680 (0.242) and 0.513 (0.248), respectively.

At the cut-off of ODR = 0.50 221 of 324 tested herds (68.2%, 95% CI: 63.0%, 73.0%) were positive for *O. ostertagi*, however there was also a great discrepancy between provinces: as many as 151 of 188 herds (80.3%, 95% CI: 74.1%, 85.4%) were positive in the north-eastern province and only 70 of 136 herds (51.5%, 95% CI: 43.1%, 59.7%) were positive in the central province.

Seven and 6 variables were initially offered to the multivariable model for *F. hepatica* (Additional file 3) and *O. ostertagi* exposure (Additional file 4), respectively. Location of a herd in the north-eastern province, longer grazing period practiced in a herd and over 50%-share of grazing grass in roughage were all positively related to the increase in exposure to both *F. hepatica* and *O. ostertagi* (Tables 2 and 3). Moreover, the use of hay or haylage as main roughage was positively related to the increase in exposure to *F. hepatica* (Table 2). The models including the aforementioned variables explained only 38.1% and 33.6% of variation of exposure to *F. hepatica* and *O. ostertagi*, respectively.

**Table 2:** Multivariable linear regression model for herd-level exposure to *Fasciola hepatica*

Risk factor	Regression coefficient (B) and standard error (SE)	95% confidence interval (95% CI) for regression coefficient	t-statistics	*p*-value
Intercept	0.18 (0.02)	–	–	–
The north-eastern province	0.21 (0.02)	0.17, 0.26	9.05	< 0.001^a^
Length of grazing period (months)	0.03 (0.01)	0.02, 0.04	6.35	< 0.001 ^a^
Grazing grass > 50% of all roughage	0.08 (0.02)	0.04, 0.13	3.38	0.001 ^a^
Hay or haylage as main roughage	0.05 (0.02)	0.01, 0.09	2.03	0.043 ^a^
Grazing on pasture > 6 h	0.06 (0.03)	−0.01, 0.12	1.67	0.095
Any grazing grass in roughage	−0.03 (0.03)	−0.08, 0.03	− 0.89	0.373
Grazing on pasture	0.03 (0.07)	−0.11, 0.17	0.38	0.706

^a^ significant risk factor for herd-level exposure to *Fasciola hepatica* at a significance level of 0.05

**Table 3:** Multivariable linear regression model for herd-level exposure to *Ostertagia ostertagi*

Risk factor	Regression coefficient (B) and standard error (SE)	95% confidence interval (95% CI) for regression coefficient	t-statistics	*p*-value
Intercept	0.42 (0.02)	–	–	–
Length of grazing period (months)	0.05 (0.01)	0.03, 0.06	8.40	< 0.001 ^b^
Grazing grass > 50% of all roughage	0.08 (0.03)	0.02, 0.13	2.60	0.010 ^b^
The north-eastern province	0.06 (0.03)	0.01, 0.11	2.24	0.026^b^
Grazing on pasture	−0.16 (0.08)	−0.31, − 0.01	−1.98	0.048^a^
Hay or haylage as main roughage	−0.02 (0.03)	−0.07, 0.04	− 0.50	0.620
Herd size	0.01 (0.01)	−0.01, 0.01	−0.11	0.912

^a^ excluded for very strong correlation with the length of grazing period.

^b^ significant risk factor for herd-level exposure to *Ostertagia ostertagi* at a significance level of 0.05.

*Fasciola* ELISA ODR and *Ostertagia* ELISA ODR were moderately positively correlated with each other in both provinces (the north-eastern province r_s_ = 0.45, *p* < 0.001, *n* = 188; the central province r_s_ = 0.58, *p* < 0.001, *n* = 136).

## Discussion

Our study shows that both *F. hepatica* and *O. ostertagi* infection is widespread in Polish cattle population, and the north-eastern part of the country appears a high-risk area for both infections.

It is difficult to compare herd-level seroprevalence of *F. hepatica* from our study with the results of studies from other countries since various methods such as coproantigen ELISA [29], f2-antigen- or cathepsin L1 antigen-based serum ELISA [17, 30, 31], post-slaughter examinations [32], or combinations of the aforementioned [33] have been used, and marked differences in their diagnostic performance have recently been demonstrated [34, 35]. Even if the same E/S ELISA had been used the authors assumed different cut-off values [7, 15, 35, 36]. We based our interpretation of *Fasciola* ELISA ODR on logical argumentation of Vercruysse and Claerebout [28] and Salimi-Bejestani et al. [8] – the cut-off of ODR = 0.27 allows to detect herds in which at least one fourth of cows is infected by *F. hepatica*, which is considered an economically significant level. Following this interpretation the north-eastern part of our country seems to be as much affected as England, Scotland and Wales (76–86%; [37]). In the Belgian and German studies [36, 38] much higher cut-off of ODR = 0.8 was used and the herd-level seroprevalences were 37% and 50–57%, respectively. If the cut-off of ODR = 0.27 had been assumed these figures perhaps would have been much higher, which implies that situation in the central part of Poland is better than in other countries of the western Europe. It seems to be more like in low-risk Scandinavian countries [7].

Median *Ostertagia* ELISA ODR in the central province of Poland (0.51, IQR from 0.34 to 0.71) was similar or slightly higher than the figures presented in two other studies carried out in the southern province [26, 27] which were 0.59 (IQR from 0.37 to 0.66) and 0.29 (IQR from 0.12 to 0.59), respectively. In the north-east median *Ostertagia* ELISA ODR (0.73, IQR from 0.57 to 0.85) was much higher than in the southern and central provinces. The same trend can be observed for the herd-level seroprevalence at the cut-off set to ODR of 0.5–30-56% in the south, 50% in the center and 80% in the north-east of the country. These results confirm that the north-east of Poland is a high-risk area. In general, situation in Poland corresponds to other European countries with the central part of the country closer to countries with moderate exposure like France, Germany, Denmark, Sweden and Netherlands, and the north-eastern part more like high-risk countries such as Belgium, UK, Ireland and Switzerland [9, 10, 17, 39]. Nevertheless, the conclusions on the economic role of *O. ostertagi* should be drawn with caution as the antigen used for the *Ostertagia* ELISA is considered to cross-react with antibodies to several other parasites including *Cooperia* spp. [40], *F. hepatica* and lungworm *Dictyocaulus viviparus* [35]. Given that these parasites differ considerably from *O. ostertagi*, *Ostertagia* ELISA is likely to cross-react with antibodies against any members of *Trichostrongyloidea* superfamily or against *Paramphistomidae* family (rumen flukes). In our study quantitative results of ELISAs for *F. hepatica* and *O. ostertagi* were also mutually correlated which may at least partly be explained by overlapping risk factors for both infections, however the role of cross-reactions can neither be excluded nor reliably assessed. Similar doubts have also been raised by other authors [17, 36]. Therefore, *Ostertagia* ELISA may have overestimated the true level of infestation with *O. ostertagi* in Polish cattle herds. Nevertheless, our results are comparable with studies carried out in other regions as the same ELISA has been used worldwide.

All included risk factors were more often encountered in the north-eastern province, which might have accounted for the higher prevalence of both infections. Nevertheless, the location of a cattle herd in this region still proved to be an independent risk factor for both infections when the model was adjusted by all the other risk factors. This likely rises from climatic differences between central and north-eastern Poland, mainly higher average monthly rainfalls in the spring and early summer in the north Poland [41], which has been previously shown to explain a considerable part of variation of *F. hepatica* occurrence [42–44].

Risk factors identified in our study generally match to these already known. Length of grazing period and high proportion of grazing grass in roughage have been evidenced to increase the risk of exposure to *F. hepatica* and *O. ostertagi* [9–11, 15, 17] simply because they are pasture-borne parasites. The univariable analysis indicated that herd size was negatively linked to the risk of exposure to *O. ostertagi*, which was consistent with many previous studies [7, 9–11], however, this factor did not hold in the multivariable analysis. This may result from the fact that Polish herds are small compared to those included in most of other studies. On the other hand, in the recent Swiss study [17], where most of herds were similarly small, herd size proved to increase the risk of exposure to *O. ostertagi*, which indicates that the role of herd size in the epidemiology of *O. ostertagi* is unclear and any conclusion should be drawn very cautiously. Contrary to some previous studies [11, 17] we did not include milk yield in our risk analysis as reduced milk yield is a consequence of more intensive exposure to the parasites [2, 37, 45] rather than a factor affecting the exposure. The only thing not mentioned before is the fact that using hay or haylage as main roughage increases risk of exposure to *F. hepatica*, when compared to corn silage, even when no pasture or fresh grass is available. This likely results from the presence of infective encysted metacercariae in forages harvested from meadows, and their resistance to the process of drying or fermentation involved in production of hay or silages. While it is known that metacercariae remain viable and infective in drying hay for at least several months, haylage has been considered as an unfavorable environment for metacercariae, which sustains their viability for less than 3 weeks [46]. Our observation seems to contradict this statement. Most likely explanation is that the viability of metacercariae depends on the quality of fermentation process. The process of haylage preparation in field conditions simply may not be as careful as it was in the experimental study [46], which allows metacercariae to remain viable and infective. Corn silage is safer perhaps because environmental conditions on corn fields are less favorable for metacerkariae survival than on grass pastures [47]. Moreover, corn for silage is harvested at considerably higher cutting height (usually more than 20 cm from the ground) than hay for haylage (8–10 cm). This may reduce the risk of inclusion of metacercariae in the roughage, as they have been shown to encyst mainly on the bottom parts of plants [48, 49].

The main shortcoming of our study is the lack of knowledge of climate (e.g. rainfalls, temperatures) and environmental conditions (e.g. soil humidity on pastures) on farms included in the study, as well as low refinement of farm management factors (i.e. type of pasture and water source utilized, exact share of grazing grass in diet, turnover of calves and heifers, stocking density etc.), which make the risk analysis quite superficial (which is highlighted by the low amount of variation explained by the models), in particular when compared with thorough risk analyzes published in recent years [30, 32, 37]. However, we mainly aimed to characterize epidemiological situation of *F. hepatica* and *O. ostertagi* in our country, especially in view of scarcity of currently available data not only from Poland but also from our eastern neighbors. Risk analysis was rather about to confirm that factors commonly considered as linked to the occurrence of these infections play their role in Poland as well, than to find any new, so far uncovered, ones. Furthermore, herds were not randomly enrolled in this study so results should be extrapolated with caution. We hope that using lists of collaborators of main dairies and enrolling quite high proportion of herds from each province (5–10%) at least partially compensates for lack of randomness.

## Conclusions

Both *F. hepatica* and *O. ostertagi* are widespread in cattle population in Poland, however their occurrence at a herd-level varies between different regions of Poland. This diversity can only partly be explained by different herd management, and appears linked to environmental and climate conditions typical for these regions. Our study provides basic knowledge of the epidemiological situation in the country, which is essential for effective control of those two parasitic infections in Polish cattle population.

## Additional files

Additional file 1: Clean copy of the questionnaire used in this study. The questionnaire was developed specifically for this study. (DOCX 21 kb)

Additional file 2: Detailed data regarding cattle herds enrolled in the study. Detailed data regarding cattle herds enrolled in the study including province, optical density ratio (ODR) in *Fasciola hepatica* ELISA, result of *Fasciola hepatica* ELISA according to the cut-off of 0.270, optical density ratio (ODR) in *Ostertagia ostertagi* ELISA, result of *Ostertagia ostertagi* ELISA according to the cut-off of 0.50, herd size given as the number of adult cows (> 24 month-old), average milk yield per lactation in kg, grazing policy classed into four categories (no pasture, grazing for 6 h a day, for 12 h a day, for 24 h), grazing period length in months, main roughage used in the herd (corn silage, haylage, hay), and proportion of grazing grass in diet classed into three categories (no grazing grass, < 50%, > 50%). (DOCX 85 kb)

Additional file 3: Univariable analysis of herd-level risk factors for *Fasciola hepatica* exposure. Descriptive statistics and results of univariable statistical analyses comparing *Fasciola hepatica* bulk-tank milk (BTM) optical density ratio (ODR) between cattle herds with different characteristics. (DOCX 20 kb)

Additional file 4: Univariable analysis of herd-level risk factors for *Ostertagia ostertagi* exposure. Descriptive statistics and results of univariable statistical analyses comparing *Ostertagia ostertagi* bulk-tank milk (BTM) optical density ratio (ODR) between cattle herds with different characteristics. (DOCX 20 kb)

## Abbreviations

BTM: Bulk-tank milk
CI: Confidence interval
IQR: Interquartile range
M: Desired precision of the estimation
n: Sample size required
OD: Optical density
ODR: Optical density ratio
r_s_: Spearman’s rank-ordered correlation coefficient
σ^2^: Variance in the population
