# Supplementary material for: Herd-level seroprevalence of Fasciola hepatica and Ostertagia ostertagi infection in dairy cattle population in the central and northeastern Poland
Source: BMC Vet Res. 2018 Apr 17;14:131. doi: 10.1186/s12917-018-1455-7 (PMC5905167; doi:10.1186/s12917-018-1455-7)
Supplement: Supplementary file 1 — Clean copy of the questionnaire used in this study. The questionnaire was developed specifically for this study. (DOCX 21 kb) [file 12917_2018_1455_MOESM1_ESM.docx]

Additional file 1. Clean copy of the questionnaire used in this study

| Address: |  |
| --- | --- |
| Date of sample collection (dd/mm/yyyy): ___/___/20__ |  |

Information regarding the herd:

1. Number of cows currently in the herd:

- Adult (>24 month-old) cows: _____________
- Heifers (>6 month-old but prior to calving): __________
- Calves (<6 month-old): __________

1. Average individual milk yield per lactation [kg]: _____________
2. Grazing policy:

□ no grazing

□ 6 hours per day

□ 12 hours per day

□ 24 hours on pasture

1. Part of the year for which cows are grazed (in months): from ___________ through __________
2. What is the main roughage used in the herd:

□ hay

□ haylage

□ corn silage

□ others _______________

1. Approximate proportion of grazing grass in diet (in %): __________
2. Number of calves / heifers / adult cows dewormed in the last year: _______ / _______ / ________
3. Number of times calves / heifers / adult cows have been dewormed in the last year: ___/___/___
4. Drug used for deworming of calves / heifers / adult cows: _______ / _______ / ________
5. Has the herd ever been screened for internal parasites?

□ Yes.

- When for the last time: ___________
- What material was used:

□ individual fecal samples

□ pooled fecal samples

□ individual blood samples

□ BTM

□ No

1. Which parasites have ever been found in the herd?

□ *Fasciola hepatica* – when _______________

□ gastrointestinal worms – when _______________

□ lung worms – when _______________

□ *Coccidia* – when _______________
