# Supplementary material for: Herd-level seroprevalence of Fasciola hepatica and Ostertagia ostertagi infection in dairy cattle population in the central and northeastern Poland
Source: BMC Vet Res. 2018 Apr 17;14:131. doi: 10.1186/s12917-018-1455-7 (PMC5905167; doi:10.1186/s12917-018-1455-7)
Supplement: Supplementary file 2 — Detailed data regarding cattle herds enrolled in the study. Detailed data regarding cattle herds enrolled in the study including province, optical density ratio (ODR) in Fasciola hepatica ELISA, result of Fasciola hepatica ELISA according to the cut-off of 0.270, optical density ratio (ODR) in Ostertagia ostertagi ELISA, result of Ostertagia ostertagi ELISA according to the cut-off of 0.50, herd size given as the number of adult cows (> 24 month-old), average milk yield per lactation in kg, grazing policy classed into four categories (no pasture, grazing for 6 h a day, for 12 h a day, for 24 h), grazing period length in months, main roughage used in the herd (corn silage, haylage, hay), and proportion of grazing grass in diet classed into three categories (no grazing grass, < 50%, > 50%). (DOCX 85 kb) [file 12917_2018_1455_MOESM2_ESM.docx]

Additional file 2. Detailed data regarding cattle herds enrolled in the study

| Herd number | Province | *Fasciola hepatica* optical density ratio (ODR) | *Fasciola hepatica* cut-off of 0.270 | *Ostertagia ostertagi* optical density ratio (ODR) | *Ostertagia ostertagi* cut-off of 0.50 | Herd size | Average milk yield per lactation [kg] | Grazing policy | Grazing period length in months | Main roughage | Proportion of grazing grass in diet |
| --- | --- | --- | --- | --- | --- | --- | --- | --- | --- | --- | --- |
| 1 | Łódzkie | 0.002 | Negative |  |  | 42 | 6100 | No grazing | 0 | corn silage | 0% |
| 2 | Łódzkie | 0.062 | Negative |  |  | 19 | 6000 | No grazing | 0 | haylage | 0% |
| 3 | Łódzkie | 0.101 | Negative |  |  | 28 | 4400 | No grazing | 0 | corn silage | 0% |
| 4 | Łódzkie | 0.191 | Negative |  |  | 18 | 5800 | No grazing | 0 | corn silage | 0% |
| 5 | Łódzkie | 0.185 | Negative |  |  | 12 | 4700 | No grazing | 0 | corn silage | 0% |
| 6 | Łódzkie | 0.093 | Negative |  |  | 14 | 5800 | No grazing | 0 | corn silage | 0% |
| 7 | Łódzkie | 0.163 | Negative | 0.26 | Negative | 25 | 5000 | No grazing | 0 | corn silage | 0% |
| 8 | Łódzkie | 0.357 | Positive | 0.93 | Positive | 10 | 5000 | 12h | 6 | haylage | >50% |
| 9 | Łódzkie | 0.071 | Negative |  |  | 28 | 5800 | No grazing | 0 | corn silage | 0% |
| 10 | Łódzkie | 0.073 | Negative |  |  | 10 | 3500 | No grazing | 0 | haylage | <50% |
| 11 | Łódzkie | 0.043 | Negative |  |  | 10 | 5000 | No grazing | 0 | hay | >50% |
| 12 | Łódzkie | 0.065 | Negative |  |  | 51 | 7500 | No grazing | 0 | corn silage | <50% |
| 13 | Łódzkie | 0.883 | Positive | 0.82 | Positive | 14 | 5550 | 12h | 5 | corn silage | <50% |
| 14 | Łódzkie | 0.072 | Negative |  |  | 38 | 5500 | No grazing | 0 | corn silage | <50% |
| 15 | Łódzkie | 0.081 | Negative |  |  | 15 | 3800 | No grazing | 0 | haylage | <50% |
| 16 | Łódzkie | 0.418 | Positive |  |  | 45 | 5700 | No grazing | 0 | corn silage | 0% |
| 17 | Łódzkie | 0.038 | Negative |  |  | 40 | 5000 | No grazing | 0 | corn silage | 0% |
| 18 | Łódzkie | 0.132 | Negative |  |  | 11 | 4900 | No grazing | 0 | corn silage | <50% |
| 19 | Łódzkie | 0.374 | Positive |  |  | 23 | 4800 | No grazing | 0 | haylage | 0% |
| 20 | Łódzkie | 0.123 | Negative | 0.09 | Negative | 12 | 5000 | No grazing | 0 | corn silage | 0% |
| 21 | Łódzkie | 0.243 | Negative | 0.60 | Positive | 16 | 5000 | 6h | 6 | corn silage | <50% |
| 22 | Łódzkie | 0.078 | Negative | 0.15 | Negative | 20 | 7000 | 12h | 5 | corn silage | <50% |
| 23 | Łódzkie | 0.080 | Negative |  |  | 16 | 6800 | No grazing | 0 | corn silage | <50% |
| 24 | Łódzkie | 0.157 | Negative | 0.34 | Negative | 15 | 7000 | No grazing | 0 | corn silage | <50% |
| 25 | Łódzkie | 0.206 | Negative | 0.14 | Negative | 21 | 6500 | No grazing | 0 | haylage | 0% |
| 26 | Łódzkie | 0.114 | Negative | 0.36 | Negative | 50 | 6700 | No grazing | 0 | corn silage | <50% |
| 27 | Łódzkie | 0.525 | Positive | 0.36 | Negative | 40 | 8000 | No grazing | 0 | haylage | 0% |
| 28 | Łódzkie | 0.626 | Positive | 0.72 | Positive | 18 | 5000 | 12h | 7 | corn silage | <50% |
| 29 | Łódzkie | 0.098 | Negative | 0.49 | Negative | 18 | 6500 | No grazing | 0 | corn silage | <50% |
| 30 | Łódzkie | 0.617 | Positive | 0.42 | Negative | 24 | 4800 | 12h | 5 | corn silage | <50% |
| 31 | Łódzkie | 0.715 | Positive | 0.79 | Positive | 20 | 6300 | 12h | 5 | haylage | >50% |
| 32 | Łódzkie | 0.365 | Positive | 0.78 | Positive | 50 | 6200 | No grazing | 0 | corn silage | <50% |
| 33 | Łódzkie | 0.186 | Negative | 0.42 | Negative | 20 | 4000 | 6h | 3 | corn silage | <50% |
| 34 | Łódzkie | 0.337 | Positive | 0.23 | Negative | 40 | 4500 | No grazing | 0 | corn silage | 0% |
| 35 | Łódzkie | 0.172 | Negative | 0.51 | Positive | 17 | 5200 | 12h | 6 | corn silage | <50% |
| 36 | Łódzkie | 0.144 | Negative | 0.61 | Positive | 41 | 6000 | No grazing | 0 | corn silage | <50% |
| 37 | Łódzkie | 0.094 | Negative | 0.41 | Negative | 18 | 7500 | No grazing | 0 | corn silage | 0% |
| 38 | Łódzkie | 0.218 | Negative | 0.56 | Positive | 35 | 7900 | 12h | 5 | corn silage | <50% |
| 39 | Łódzkie | 0.135 | Negative | 0.23 | Negative | 18 | 7700 | No grazing | 0 | corn silage | 0% |
| 40 | Łódzkie | 0.404 | Positive | 0.65 | Positive | 12 | 6100 | 6h | 5 | corn silage | <50% |
| 41 | Łódzkie | 0.134 | Negative | 0.30 | Negative | 18 | 5000 | No grazing | 0 | corn silage | 0% |
| 42 | Łódzkie | 0.116 | Negative | 0.63 | Positive | 12 | 4500 | 6h | 5 | corn silage | >50% |
| 43 | Łódzkie | 0.262 | Negative | 0.55 | Positive | 17 | 4500 | 6h | 5 | corn silage | <50% |
| 44 | Łódzkie | 0.129 | Negative |  |  | 50 | 6000 | No grazing | 0 | corn silage | <50% |
| 45 | Łódzkie | 0.170 | Negative | 0.64 | Positive | 25 | 5800 | 6h | 5 | corn silage | <50% |
| 46 | Łódzkie | 0.119 | Negative | 0.25 | Negative | 16 | 5000 | No grazing | 0 | corn silage | <50% |
| 47 | Łódzkie | 0.438 | Positive | 0.72 | Positive | 21 | 9100 | 12h | 4 | corn silage | <50% |
| 48 | Łódzkie | 0.509 | Positive | 0.61 | Positive | 15 | 7000 | No grazing | 0 | corn silage | 0% |
| 49 | Łódzkie | 0.104 | Negative | 0.44 | Negative | 100 | 7500 | No grazing | 0 | corn silage | 0% |
| 50 | Łódzkie | 0.887 | Positive | 0.81 | Positive | 21 | 6500 | 12h | 5 | corn silage | <50% |
| 51 | Łódzkie | 0.166 | Negative | 0.72 | Positive | 20 | 4500 | No grazing | 0 | corn silage | 0% |
| 52 | Łódzkie | 0.119 | Negative | 0.27 | Negative | 14 | 6800 | No grazing | 0 | corn silage | 0% |
| 53 | Łódzkie | 0.087 | Negative | 0.35 | Negative | 22 | 8000 | No grazing | 0 | corn silage | <50% |
| 54 | Łódzkie | 0.196 | Negative | 0.14 | Negative | 14 | 3000 | No grazing | 0 | corn silage | <50% |
| 55 | Łódzkie | 0.375 | Positive | 0.67 | Positive | 19 | 8900 | No grazing | 0 | corn silage | 0% |
| 56 | Łódzkie | 0.337 | Positive | 0.25 | Negative | 18 | 7000 | No grazing | 0 | corn silage | <50% |
| 57 | Łódzkie | 0.679 | Positive | 0.70 | Positive | 18 | 5000 | 12h | 6 | corn silage | <50% |
| 58 | Łódzkie | 0.286 | Positive | 0.01 | Negative | 21 | 6000 | 12h | 5 | corn silage | <50% |
| 59 | Łódzkie | 0.972 | Positive | 0.91 | Positive | 30 | 4700 | 12h | 6 | corn silage | <50% |
| 60 | Łódzkie | 0.083 | Negative | 0.35 | Negative | 10 | 7300 | No grazing | 0 | corn silage | 0% |
| 61 | Łódzkie | 0.066 | Negative | 0.01 | Negative | 15 | 6500 | No grazing | 0 | corn silage | <50% |
| 62 | Łódzkie | 0.097 | Negative | 0.51 | Positive | 15 | 6000 | No grazing | 0 | haylage | 0% |
| 63 | Łódzkie | 0.361 | Positive | 0.65 | Positive | 15 | 6000 | 12h | 6 | corn silage | <50% |
| 64 | Łódzkie | 0.112 | Negative | 0.11 | Negative | 25 | 6000 | No grazing | 0 | haylage | 0% |
| 65 | Łódzkie | 0.064 | Negative |  |  | 38 | 7500 | No grazing | 0 | corn silage | 0% |
| 66 | Łódzkie | 0.364 | Positive | 0.47 | Negative | 15 | 8320 | 12h | 5 | haylage | <50% |
| 67 | Łódzkie | 0.104 | Negative | 0.09 | Negative | 11 | 5000 | 6h | 1 | corn silage | <50% |
| 68 | Łódzkie | 0.093 | Negative | 0.17 | Negative | 18 | 6000 | No grazing | 0 | corn silage | 0% |
| 69 | Łódzkie | 0.244 | Negative | 0.58 | Positive | 21 | 5550 | 6h | 5 | haylage | <50% |
| 70 | Łódzkie | 0.133 | Negative | 0.32 | Negative | 14 | 6000 | 12h | 6 | corn silage | <50% |
| 71 | Łódzkie | 0.120 | Negative | 0.37 | Negative | 12 | 4500 | No grazing | 0 | corn silage | <50% |
| 72 | Łódzkie | 0.935 | Positive | 0.49 | Negative | 14 | 4000 | 12h | 5 | hay | <50% |
| 73 | Łódzkie | 0.529 | Positive | 0.82 | Positive | 19 | 7200 | 12h | 6 | corn silage | <50% |
| 74 | Łódzkie | 0.231 | Negative | 0.16 | Negative | 25 | 7500 | No grazing | 0 | corn silage | 0% |
| 75 | Łódzkie | 0.106 | Negative | 0.45 | Negative | 17 | 5000 | No grazing | 0 | corn silage | 0% |
| 76 | Łódzkie | 0.095 | Negative | 0.40 | Negative | 24 | 7000 | No grazing | 0 | corn silage | 0% |
| 77 | Łódzkie | 0.090 | Negative | 0.48 | Negative | 35 | 6000 | No grazing | 0 | corn silage | <50% |
| 78 | Łódzkie | 0.112 | Negative | 0.48 | Negative | 25 | 6000 | 6h | 5 | corn silage | <50% |
| 79 | Łódzkie | 0.058 | Negative |  |  | 31 | 5800 | No grazing | 0 | corn silage | 0% |
| 80 | Łódzkie | 0.514 | Positive | 0.39 | Negative | 10 | 4800 | No grazing | 0 | haylage | <50% |
| 81 | Łódzkie | 0.595 | Positive |  |  | 14 | 4200 | No grazing | 0 | corn silage | 0% |
| 82 | Łódzkie | 0.104 | Negative |  |  | 14 | 4200 | No grazing | 0 | corn silage | 0% |
| 83 | Łódzkie | 0.072 | Negative |  |  | 17 | 4300 | No grazing | 0 | corn silage | 0% |
| 84 | Łódzkie | 0.122 | Negative | 0.54 | Positive | 11 | 5000 | No grazing | 0 | haylage | >50% |
| 85 | Łódzkie | 0.143 | Negative |  |  | 28 | 7500 | 12h | 2 | haylage | >50% |
| 86 | Łódzkie | 0.334 | Positive | 0.01 | Negative | 17 | 6000 | No grazing | 0 | haylage | >50% |
| 87 | Łódzkie | 0.161 | Negative | 0.39 | Negative | 10 | 5000 | No grazing | 0 | corn silage | 0% |
| 88 | Łódzkie | 0.296 | Positive |  |  | 12 | 4800 | No grazing | 0 | corn silage | 0% |
| 89 | Łódzkie | 0.063 | Negative |  |  | 29 | 4200 | No grazing | 0 | corn silage | 0% |
| 90 | Łódzkie | 0.019 | Negative |  |  | 42 | 5000 | No grazing | 0 | corn silage | 0% |
| 91 | Łódzkie | 0.134 | Negative | 0.42 | Negative | 12 | 4600 | No grazing | 0 | corn silage | 0% |
| 92 | Łódzkie | 0.136 | Negative | 0.38 | Negative | 12 | 4900 | No grazing | 0 | corn silage | <50% |
| 93 | Łódzkie | 0.351 | Positive | 0.71 | Positive | 16 | 4500 | No grazing | 0 | corn silage | 0% |
| 94 | Łódzkie | 0.333 | Positive | 0.56 | Positive | 13 | 5100 | No grazing | 0 | corn silage | 0% |
| 95 | Łódzkie | 0.269 | Negative | 0.64 | Positive | 29 | 4800 | No grazing | 0 | corn silage | 0% |
| 96 | Łódzkie | 0.199 | Negative | 0.47 | Negative | 12 | 5200 | No grazing | 0 | corn silage | 0% |
| 97 | Łódzkie | 0.388 | Positive |  |  | 25 | 5550 | No grazing | 0 | haylage | 0% |
| 98 | Łódzkie | 0.206 | Negative |  |  | 28 | 5000 | No grazing | 0 | corn silage | 0% |
| 99 | Łódzkie | 0.497 | Positive | 1.04 | Positive | 15 | 6000 | 12h | 5 | corn silage | <50% |
| 100 | Łódzkie | 0.019 | Negative |  |  | 24 | 6400 | No grazing | 0 | corn silage | 0% |
| 101 | Łódzkie | 0.146 | Negative | 0.65 | Positive | 10 | 4000 | No grazing | 0 | hay | 0% |
| 102 | Łódzkie | 0.202 | Negative |  |  | 16 | 4000 | No grazing | 0 | haylage | <50% |
| 103 | Łódzkie | 0.512 | Positive |  |  | 16 | 4300 | No grazing | 0 | corn silage | 0% |
| 104 | Łódzkie | 0.211 | Negative |  |  | 18 | 4600 | No grazing | 0 | corn silage | 0% |
| 105 | Łódzkie | 0.213 | Negative | 0.64 | Positive | 24 | 4800 | No grazing | 0 | corn silage | 0% |
| 106 | Łódzkie | 0.216 | Negative | 0.57 | Positive | 15 | 4200 | No grazing | 0 | corn silage | 0% |
| 107 | Łódzkie | 0.120 | Negative | 0.25 | Negative | 10 | 4400 | No grazing | 0 | haylage | 0% |
| 108 | Łódzkie | 0.301 | Positive | 0.26 | Negative | 12 | 4300 | No grazing | 0 | corn silage | 0% |
| 109 | Łódzkie | 0.084 | Negative |  |  | 18 | 5200 | No grazing | 0 | corn silage | 0% |
| 110 | Łódzkie | 0.106 | Negative |  |  | 12 | 4200 | 6h | 5 | haylage | <50% |
| 111 | Łódzkie | 0.208 | Negative |  |  | 48 | 5800 | No grazing | 0 | corn silage | 0% |
| 112 | Łódzkie | 0.129 | Negative |  |  | 22 | 3600 | 6h | 4 | corn silage | <50% |
| 113 | Łódzkie | 0.055 | Negative |  |  | 59 | 5300 | No grazing | 0 | corn silage | 0% |
| 114 | Łódzkie | 0.060 | Negative |  |  | 14 | 4000 | No grazing | 0 | haylage | <50% |
| 115 | Łódzkie | 0.149 | Negative |  |  | 20 | 4500 | No grazing | 0 | corn silage | <50% |
| 116 | Łódzkie | 0.079 | Negative |  |  | 35 | 5000 | No grazing | 0 | corn silage | 0% |
| 117 | Łódzkie | 0.113 | Negative |  |  | 12 | 5550 | 6h | 5 | haylage | <50% |
| 118 | Łódzkie | 0.128 | Negative | 0.46 | Negative | 33 | 4800 | No grazing | 0 | haylage | 0% |
| 119 | Łódzkie | 0.589 | Positive | 0.22 | Negative | 13 | 4800 | 12h | 5 | corn silage | <50% |
| 120 | Łódzkie | 0.137 | Negative |  |  | 12 | 5000 | 12h | 4 | corn silage | <50% |
| 121 | Łódzkie | 0.069 | Negative |  |  | 36 | 4900 | No grazing | 0 | corn silage | 0% |
| 122 | Łódzkie | 0.077 | Negative |  |  | 11 | 4800 | No grazing | 0 | corn silage | <50% |
| 123 | Łódzkie | 0.113 | Negative |  |  | 14 | 4300 | No grazing | 0 | corn silage | <50% |
| 124 | Łódzkie | 0.347 | Positive | 0.88 | Positive | 24 | 4800 | No grazing | 0 | corn silage | 0% |
| 125 | Łódzkie | 0.072 | Negative | 0.04 | Negative | 14 | 4500 | No grazing | 0 | corn silage | 0% |
| 126 | Łódzkie | 0.045 | Negative |  |  | 37 | 5500 | No grazing | 0 | corn silage | <50% |
| 127 | Łódzkie | 0.401 | Positive |  |  | 29 | 4800 | No grazing | 0 | corn silage | <50% |
| 128 | Łódzkie | 0.115 | Negative |  |  | 20 | 4900 | No grazing | 0 | corn silage | 0% |
| 129 | Łódzkie | 0.046 | Negative |  |  | 22 | 4200 | No grazing | 0 | corn silage | 0% |
| 130 | Łódzkie | 0.432 | Positive |  |  | 10 | 4200 | No grazing | 0 | corn silage | 0% |
| 131 | Łódzkie | 0.026 | Negative |  |  | 37 | 5400 | No grazing | 0 | corn silage | <50% |
| 132 | Łódzkie | 0.056 | Negative |  |  | 12 | 5200 | No grazing | 0 | corn silage | 0% |
| 133 | Łódzkie | 0.047 | Negative |  |  | 45 | 5600 | No grazing | 0 | corn silage | 0% |
| 134 | Łódzkie | 0.066 | Negative |  |  | 10 | 4700 | No grazing | 0 | corn silage | <50% |
| 135 | Łódzkie | 0.082 | Negative |  |  | 22 | 4000 | No grazing | 0 | corn silage | 0% |
| 136 | Łódzkie | 0.069 | Negative |  |  | 20 | 5000 | No grazing | 0 | corn silage | <50% |
| 137 | Łódzkie | 0.078 | Negative |  |  | 18 | 5800 | No grazing | 0 | corn silage | 0% |
| 138 | Łódzkie | 0.056 | Negative |  |  | 23 | 4200 | No grazing | 0 | corn silage | 0% |
| 139 | Łódzkie | 0.502 | Positive |  |  | 17 | 3200 | No grazing | 0 | corn silage | <50% |
| 140 | Łódzkie | 0.347 | Positive | 0.95 | Positive | 10 | 4800 | 12h | 5 | corn silage | <50% |
| 141 | Łódzkie | 0.094 | Negative |  |  | 16 | 4600 | No grazing | 0 | corn silage | 0% |
| 142 | Łódzkie | 0.185 | Negative | 0.64 | Positive | 16 | 4500 | No grazing | 0 | corn silage | 0% |
| 143 | Łódzkie | 0.207 | Negative |  |  | 12 | 4200 | 6h | 5 | haylage | >50% |
| 144 | Łódzkie | 0.098 | Negative | 0.01 | Negative | 28 | 4800 | No grazing | 0 | haylage | 0% |
| 145 | Łódzkie | 0.854 | Positive | 0.37 | Negative | 30 | 4600 | No grazing | 0 | corn silage | 0% |
| 146 | Łódzkie | 0.799 | Positive |  |  | 13 | 5800 | No grazing | 0 | haylage | 0% |
| 147 | Łódzkie | 0.409 | Positive | 0.68 | Positive | 15 | 4600 | No grazing | 0 | corn silage | 0% |
| 148 | Łódzkie | 0.125 | Negative | 0.47 | Negative | 12 | 5200 | No grazing | 0 | corn silage | <50% |
| 149 | Łódzkie | 0.178 | Negative |  |  | 12 | 4700 | No grazing | 0 | corn silage | 0% |
| 150 | Łódzkie | 0.064 | Negative |  |  | 22 | 4800 | No grazing | 0 | corn silage | <50% |
| 151 | Łódzkie | 0.092 | Negative | 0.29 | Negative | 33 | 4900 | No grazing | 0 | corn silage | 0% |
| 152 | Łódzkie | 0.361 | Positive | 0.78 | Positive | 15 | 4300 | No grazing | 0 | corn silage | 0% |
| 153 | Łódzkie | 0.065 | Negative |  |  | 18 | 5800 | No grazing | 0 | haylage | <50% |
| 154 | Łódzkie | 0.262 | Negative | 0.50 | Positive | 13 | 4800 | No grazing | 0 | corn silage | 0% |
| 155 | Łódzkie | 0.176 | Negative |  |  | 16 | 4800 | No grazing | 0 | corn silage | <50% |
| 156 | Łódzkie | 0.333 | Positive |  |  | 18 | 4500 | No grazing | 0 | haylage | 0% |
| 157 | Podlaskie | 0.611 | Positive |  |  | 16 | 5700 | 12h | 3 | haylage | <50% |
| 158 | Podlaskie | 0.324 | Positive |  |  | 38 | 6000 | No grazing | 0 | corn silage | <50% |
| 159 | Podlaskie | 0.114 | Negative |  |  | 21 | 7200 | 12h | 6 | corn silage | <50% |
| 160 | Podlaskie | 0.231 | Negative |  |  | 25 | 5500 | No grazing | 0 | haylage | 0% |
| 161 | Podlaskie | 0.334 | Positive |  |  | 24 | 6000 | 12h | 4 | corn silage | <50% |
| 162 | Podlaskie | 0.584 | Positive |  |  | 30 | 8100 | No grazing | 0 | corn silage | 0% |
| 163 | Podlaskie | 0.561 | Positive |  |  | 13 | 4000 | 12h | 5 | haylage | >50% |
| 164 | Podlaskie | 0.562 | Positive |  |  | 22 | 6500 | No grazing | 0 | corn silage | <50% |
| 165 | Podlaskie | 0.169 | Negative |  |  | 30 | 6900 | No grazing | 0 | corn silage | 0% |
| 166 | Podlaskie | 0.524 | Positive |  |  | 12 | 5100 | 12h | 5 | haylage | >50% |
| 167 | Podlaskie | 0.090 | Negative |  |  | 21 | 8000 | No grazing | 0 | haylage | <50% |
| 168 | Podlaskie | 0.186 | Negative |  |  | 21 | 7200 | No grazing | 0 | corn silage | 0% |
| 169 | Podlaskie | 0.287 | Positive |  |  | 20 | 6100 | No grazing | 0 | haylage | 0% |
| 170 | Podlaskie | 0.084 | Negative |  |  | 25 | 7900 | No grazing | 0 | corn silage | 0% |
| 171 | Podlaskie | 0.413 | Positive |  |  | 17 | 5500 | No grazing | 0 | haylage | 0% |
| 172 | Podlaskie | 0.431 | Positive |  |  | 16 | 5500 | 12h | 5 | corn silage | <50% |
| 173 | Podlaskie | 0.091 | Negative |  |  | 36 | 6700 | No grazing | 0 | corn silage | 0% |
| 174 | Podlaskie | 0.423 | Positive |  |  | 33 | 6500 | No grazing | 0 | corn silage | 0% |
| 175 | Podlaskie | 0.462 | Positive |  |  | 11 | 5800 | 6h | 5 | corn silage | >50% |
| 176 | Podlaskie | 0.062 | Negative |  |  | 44 | 7500 | No grazing | 0 | haylage | 0% |
| 177 | Podlaskie | 0.555 | Positive |  |  | 18 | 6200 | No grazing | 0 | haylage | 0% |
| 178 | Podlaskie | 0.552 | Positive |  |  | 32 | 6000 | 6h | 5 | haylage | <50% |
| 179 | Podlaskie | 0.118 | Negative |  |  | 12 | 4500 | No grazing | 0 | corn silage | <50% |
| 180 | Podlaskie | 0.299 | Positive |  |  | 18 | 4600 | 6h | 4 | corn silage | <50% |
| 181 | Podlaskie | 0.076 | Negative |  |  | 27 | 6500 | No grazing | 0 | corn silage | <50% |
| 182 | Podlaskie | 0.200 | Negative |  |  | 55 | 8900 | No grazing | 0 | corn silage | <50% |
| 183 | Podlaskie | 0.358 | Positive |  |  | 25 | 4600 | No grazing | 0 | corn silage | <50% |
| 184 | Podlaskie | 0.089 | Negative |  |  | 48 | 4200 | No grazing | 0 | corn silage | <50% |
| 185 | Podlaskie | 0.473 | Positive |  |  | 28 | 5500 | 6h | 4 | corn silage | <50% |
| 186 | Podlaskie | 0.556 | Positive |  |  | 14 | 5600 | No grazing | 0 | haylage | 0% |
| 187 | Podlaskie | 0.320 | Positive |  |  | 48 | 7200 | No grazing | 0 | corn silage | 0% |
| 188 | Podlaskie | 0.191 | Negative |  |  | 25 | 6200 | No grazing | 0 | haylage | 0% |
| 189 | Podlaskie | 0.313 | Positive |  |  | 45 | 5800 | No grazing | 0 | haylage | <50% |
| 190 | Podlaskie | 0.335 | Positive |  |  | 16 | 5300 | 6h | 4 | corn silage | <50% |
| 191 | Podlaskie | 0.115 | Negative |  |  | 20 | 5000 | 12h | 5 | haylage | >50% |
| 192 | Podlaskie | 0.217 | Negative |  |  | 32 | 8200 | 6h | 4 | corn silage | <50% |
| 193 | Podlaskie | 0.637 | Positive |  |  | 26 | 6500 | No grazing | 0 | haylage | 0% |
| 194 | Podlaskie | 0.103 | Negative |  |  | 32 | 6700 | No grazing | 0 | corn silage | 0% |
| 195 | Podlaskie | 0.527 | Positive |  |  | 22 | 3900 | No grazing | 0 | corn silage | <50% |
| 196 | Podlaskie | 0.080 | Negative |  |  | 18 | 4500 | No grazing | 0 | corn silage | <50% |
| 197 | Podlaskie | 0.566 | Positive |  |  | 29 | 7100 | 6h | 5 | haylage | >50% |
| 198 | Podlaskie | 0.537 | Positive |  |  | 20 | 4500 | 12h | 5 | corn silage | <50% |
| 199 | Podlaskie | 0.620 | Positive |  |  | 29 | 6000 | 12h | 5 | haylage | <50% |
| 200 | Podlaskie | 0.145 | Negative |  |  | 30 | 6800 | No grazing | 0 | corn silage | 0% |
| 201 | Podlaskie | 0.585 | Positive |  |  | 27 | 4000 | 12h | 5 | corn silage | <50% |
| 202 | Podlaskie | 0.077 | Negative |  |  | 19 | 6000 | No grazing | 0 | haylage | <50% |
| 203 | Podlaskie | 0.499 | Positive |  |  | 18 | 6200 | No grazing | 0 | corn silage | 0% |
| 204 | Podlaskie | 0.505 | Positive |  |  | 25 | 7400 | 12h | 2 | haylage | <50% |
| 205 | Podlaskie | 0.959 | Positive | 0.76 | Positive | 30 | 5000 | 6h | 5 | haylage | >50% |
| 206 | Podlaskie | 0.164 | Negative | 0.34 | Negative | 29 | 3100 | No grazing | 0 | haylage | <50% |
| 207 | Podlaskie | 0.695 | Positive |  |  | 21 | 4500 | 12h | 6 | haylage | >50% |
| 208 | Podlaskie | 0.799 | Positive | 0.65 | Positive | 35 | 7000 | 6h | 4 | haylage | <50% |
| 209 | Podlaskie | 0.552 | Positive |  |  | 17 | 5000 | 12h | 5 | haylage | <50% |
| 210 | Podlaskie | 0.887 | Positive | 0.73 | Positive | 10 | 3000 | 6h | 3 | haylage | <50% |
| 211 | Podlaskie | 0.185 | Negative |  |  | 38 | 5800 | 6h | 5 | corn silage | <50% |
| 212 | Podlaskie | 0.314 | Positive |  |  | 21 | 5800 | No grazing | 0 | corn silage | <50% |
| 213 | Podlaskie | 0.259 | Negative | 0.68 | Positive | 78 | 8300 | No grazing | 0 | haylage | 0% |
| 214 | Podlaskie | 0.393 | Positive |  |  | 20 | 5500 | No grazing | 0 | haylage | <50% |
| 215 | Podlaskie | 0.677 | Positive |  |  | 18 | 3000 | 6h | 4 | haylage | <50% |
| 216 | Podlaskie | 0.553 | Positive |  |  | 28 | 5100 | 12h | 6 | haylage | <50% |
| 217 | Podlaskie | 0.454 | Positive |  |  | 38 | 6000 | No grazing | 0 | corn silage | <50% |
| 218 | Podlaskie | 0.108 | Negative |  |  | 35 | 7400 | No grazing | 0 | corn silage | 0% |
| 219 | Podlaskie | 0.602 | Positive |  |  | 31 | 4500 | 12h | 6 | haylage | <50% |
| 220 | Podlaskie | 0.677 | Positive |  |  | 23 | 5500 | 24h | 5 | haylage | <50% |
| 221 | Podlaskie | 0.815 | Positive | 0.84 | Positive | 30 | 6000 | 12h | 5 | haylage | <50% |
| 222 | Podlaskie | 0.475 | Positive |  |  | 20 | 4600 | 6h | 4 | corn silage | <50% |
| 223 | Podlaskie | 0.214 | Negative |  |  | 17 | 5100 | 12h | 5 | haylage | <50% |
| 224 | Podlaskie | 0.105 | Negative |  |  | 16 | 6100 | No grazing | 0 | corn silage | <50% |
| 225 | Podlaskie | 0.090 | Negative |  |  | 30 | 6500 | No grazing | 0 | haylage | <50% |
| 226 | Podlaskie | 0.621 | Positive | 0.78 | Positive | 28 | 6400 | 12h | 4 | haylage | >50% |
| 227 | Podlaskie | 0.084 | Negative |  |  | 18 | 5800 | 12h | 4 | haylage | <50% |
| 228 | Podlaskie | 0.547 | Positive |  |  | 18 | 4200 | 6h | 3 | haylage | <50% |
| 229 | Podlaskie | 0.811 | Positive | 0.43 | Negative | 37 | 7000 | No grazing | 0 | corn silage | 0% |
| 230 | Podlaskie | 0.472 | Positive |  |  | 10 | 6000 | No grazing | 0 | corn silage | 0% |
| 231 | Podlaskie | 0.151 | Negative |  |  | 16 | 6100 | 6h | 4 | haylage | <50% |
| 232 | Podlaskie | 0.097 | Negative |  |  | 49 | 8300 | No grazing | 0 | haylage | 0% |
| 233 | Podlaskie | 0.120 | Negative |  |  | 14 | 4100 | 6h | 4 | corn silage | <50% |
| 234 | Podlaskie | 0.068 | Negative |  |  | 30 | 7500 | No grazing | 0 | corn silage | 0% |
| 235 | Podlaskie | 0.236 | Negative |  |  | 31 | 5700 | No grazing | 0 | haylage | 0% |
| 236 | Podlaskie | 0.911 | Positive | 0.85 | Positive | 46 | 7000 | 12h | 5 | corn silage | <50% |
| 237 | Podlaskie | 0.526 | Positive |  |  | 26 | 4800 | No grazing | 0 | corn silage | <50% |
| 238 | Podlaskie | 0.856 | Positive | 0.98 | Positive | 24 | 6000 | 12h | 5 | haylage | >50% |
| 239 | Podlaskie | 0.863 | Positive | 0.84 | Positive | 10 | 4200 | 12h | 5 | corn silage | >50% |
| 240 | Podlaskie | 0.083 | Negative |  |  | 27 | 6100 | No grazing | 0 | haylage | <50% |
| 241 | Podlaskie | 0.270 | Positive |  |  | 28 | 6300 | 6h | 4 | corn silage | <50% |
| 242 | Podlaskie | 0.524 | Positive |  |  | 26 | 4000 | No grazing | 0 | haylage | <50% |
| 243 | Podlaskie | 0.503 | Positive |  |  | 25 | 5000 | 12h | 5 | haylage | <50% |
| 244 | Podlaskie | 0.338 | Positive |  |  | 12 | 3800 | 6h | 4 | corn silage | <50% |
| 245 | Podlaskie | 0.420 | Positive |  |  | 24 | 5000 | 12h | 4 | corn silage | <50% |
| 246 | Podlaskie | 0.182 | Negative |  |  | 11 | 6000 | No grazing | 0 | haylage | 0% |
| 247 | Podlaskie | 0.237 | Negative |  |  | 35 | 5500 | 6h | 3 | corn silage | <50% |
| 248 | Podlaskie | 0.552 | Positive |  |  | 18 | 5500 | 12h | 5 | haylage | <50% |
| 249 | Podlaskie | 0.410 | Positive |  |  | 35 | 5500 | 12h | 5 | haylage | <50% |
| 250 | Podlaskie | 0.090 | Negative |  |  | 16 | 5700 | 6h | 4 | corn silage | <50% |
| 251 | Podlaskie | 0.301 | Positive |  |  | 20 | 4500 | No grazing | 0 | haylage | <50% |
| 252 | Podlaskie | 0.512 | Positive |  |  | 22 | 5000 | 12h | 4 | corn silage | <50% |
| 253 | Podlaskie | 0.556 | Positive |  |  | 34 | 6700 | 12h | 4 | haylage | <50% |
| 254 | Podlaskie | 0.548 | Positive |  |  | 20 | 5300 | 6h | 5 | haylage | <50% |
| 255 | Podlaskie | 0.567 | Positive |  |  | 12 | 4500 | 12h | 5 | haylage | <50% |
| 256 | Podlaskie | 0.439 | Positive |  |  | 23 | 4000 | No grazing | 0 | haylage | 0% |
| 257 | Podlaskie | 0.705 | Positive |  |  | 34 | 8000 | No grazing | 0 | corn silage | 0% |
| 258 | Podlaskie | 0.416 | Positive |  |  | 60 | 10200 | No grazing | 0 | corn silage | 0% |
| 259 | Podlaskie | 0.793 | Positive | 0.84 | Positive | 15 | 4700 | 12h | 6 | hay | >50% |
| 260 | Podlaskie | 0.686 | Positive | 0.96 | Positive | 14 | 3000 | 12h | 5 | hay | >50% |
| 261 | Podlaskie | 0.911 | Positive | 0.84 | Positive | 18 | 3300 | 12h | 6 | haylage | >50% |
| 262 | Podlaskie | 0.213 | Negative | 0.69 | Positive | 12 | 2700 | 12h | 6 | haylage | >50% |
| 263 | Podlaskie | 0.702 | Positive |  |  | 19 | 7000 | 6h | 5 | haylage | <50% |
| 264 | Podlaskie | 0.384 | Positive |  |  | 70 | 6500 | 24h | 8 | haylage | >50% |
| 265 | Podlaskie | 0.082 | Negative | 0.40 | Negative | 29 | 7000 | No grazing | 0 | haylage | 0% |
| 266 | Podlaskie | 0.299 | Positive | 0.39 | Negative | 32 | 8000 | 12h | 5 | haylage | <50% |
| 267 | Podlaskie | 0.607 | Positive |  |  | 25 | 5500 | 12h | 5 | haylage | >50% |
| 268 | Podlaskie | 0.270 | Positive | 0.61 | Positive | 56 | 9203 | No grazing | 0 | haylage | 0% |
| 269 | Podlaskie | 0.741 | Positive | 0.71 | Positive | 10 | 5700 | No grazing | 0 | haylage | 0% |
| 270 | Podlaskie | 0.601 | Positive |  |  | 35 | 6300 | 24h | 4 | haylage | >50% |
| 271 | Podlaskie | 0.569 | Positive |  |  | 15 | 4500 | 12h | 5 | corn silage | <50% |
| 272 | Podlaskie | 0.616 | Positive |  |  | 12 | 5100 | 12h | 5 | haylage | >50% |
| 273 | Podlaskie | 0.442 | Positive |  |  | 34 | 5000 | 24h | 5 | haylage | <50% |
| 274 | Podlaskie | 0.787 | Positive |  |  | 26 | 4200 | No grazing | 0 | haylage | 0% |
| 275 | Podlaskie | 0.839 | Positive | 0.93 | Positive | 30 | 6000 | 24h | 5 | corn silage | >50% |
| 276 | Podlaskie | 0.170 | Negative |  |  | 15 | 4000 | 12h | 5 | corn silage | >50% |
| 277 | Podlaskie | 0.696 | Positive | 0.56 | Positive | 19 | 4600 | 12h | 5 | hay | >50% |
| 278 | Podlaskie | 0.694 | Positive | 0.92 | Positive | 25 | 5550 | 12h | 6 | hay | >50% |
| 279 | Podlaskie | 0.641 | Positive |  |  | 17 | 5600 | 6h | 5 | haylage | >50% |
| 280 | Podlaskie | 0.636 | Positive |  |  | 20 | 5000 | 12h | 5 | haylage | <50% |
| 281 | Podlaskie | 0.257 | Negative |  |  | 19 | 5000 | 24h | 6 | haylage | >50% |
| 282 | Podlaskie | 0.766 | Positive |  |  | 27 | 5700 | 6h | 5 | haylage | <50% |
| 283 | Podlaskie | 0.149 | Negative | 0.30 | Negative | 26 | 6800 | No grazing | 0 | haylage | 0% |
| 284 | Podlaskie | 0.685 | Positive |  |  | 20 | 5500 | 12h | 5 | haylage | <50% |
| 285 | Podlaskie | 0.416 | Positive | 0.72 | Positive | 19 | 4500 | 12h | 5 | haylage | <50% |
| 286 | Podlaskie | 0.406 | Positive |  |  | 26 | 6000 | 24h | 5 | haylage | >50% |
| 287 | Podlaskie | 0.844 | Positive | 0.87 | Positive | 27 | 6500 | 12h | 4 | haylage | >50% |
| 288 | Podlaskie | 0.302 | Positive | 0.69 | Positive | 90 | 7000 | 12h | 5 | corn silage | <50% |
| 289 | Podlaskie | 0.231 | Negative |  |  | 12 | 5000 | 12h | 5 | haylage | >50% |
| 290 | Podlaskie | 0.078 | Negative |  |  | 75 | 8300 | No grazing | 0 | corn silage | 0% |
| 291 | Podlaskie | 0.833 | Positive | 0.81 | Positive | 13 | 3600 | 12h | 5 | hay | >50% |
| 292 | Podlaskie | 0.620 | Positive | 0.43 | Negative | 19 | 5000 | 12h | 6 | haylage | >50% |
| 293 | Podlaskie | 0.676 | Positive | 0.82 | Positive | 10 | 3500 | 12h | 6 | haylage | >50% |
| 294 | Podlaskie | 0.798 | Positive | 0.86 | Positive | 15 | 5550 | 12h | 5 | haylage | >50% |
| 295 | Podlaskie | 0.187 | Negative | 0.49 | Negative | 24 | 6200 | 12h | 5 | haylage | <50% |
| 296 | Podlaskie | 0.832 | Positive | 0.76 | Positive | 40 | 5600 | 12h | 5 | corn silage | <50% |
| 297 | Podlaskie | 0.595 | Positive | 0.76 | Positive | 14 | 3000 | 24h | 6 | haylage | >50% |
| 298 | Podlaskie | 0.570 | Positive |  |  | 18 | 4500 | 12h | 5 | haylage | <50% |
| 299 | Podlaskie | 0.422 | Positive |  |  | 32 | 6200 | 12h | 3 | haylage | >50% |
| 300 | Podlaskie | 0.157 | Negative |  |  | 17 | 6500 | 24h | 5 | haylage | <50% |
| 301 | Podlaskie | 0.195 | Negative | 0.53 | Positive | 22 | 4500 | 24h | 5 | haylage | >50% |
| 302 | Podlaskie | 0.665 | Positive |  |  | 42 | 5200 | 24h | 6 | haylage | <50% |
| 303 | Podlaskie | 0.773 | Positive | 0.60 | Positive | 42 | 6500 | 12h | 3 | haylage | >50% |
| 304 | Podlaskie | 0.559 | Positive | 0.32 | Negative | 90 | 7500 | No grazing | 0 | corn silage | 0% |
| 305 | Podlaskie | 0.467 | Positive | 0.20 | Negative | 32 | 5500 | No grazing | 0 | haylage | 0% |
| 306 | Podlaskie | 0.847 | Positive | 0.79 | Positive | 28 | 5000 | 24h | 6 | haylage | >50% |
| 307 | Podlaskie | 0.360 | Positive | 0.12 | Negative | 21 | 2900 | No grazing | 0 | haylage | 0% |
| 308 | Podlaskie | 0.948 | Positive | 0.95 | Positive | 24 | 5000 | 12h | 5 | haylage | <50% |
| 309 | Podlaskie | 0.811 | Positive | 0.83 | Positive | 29 | 3800 | 12h | 6 | hay | >50% |
| 310 | Podlaskie | 0.822 | Positive | 0.98 | Positive | 17 | 3400 | 12h | 6 | haylage | >50% |
| 311 | Podlaskie | 0.923 | Positive | 0.89 | Positive | 30 | 6600 | 12h | 4 | haylage | >50% |
| 312 | Podlaskie | 0.545 | Positive |  |  | 13 | 4000 | 12h | 4 | corn silage | >50% |
| 313 | Podlaskie | 0.425 | Positive |  |  | 17 | 5550 | 12h | 5 | haylage | >50% |
| 314 | Podlaskie | 0.234 | Negative | 0.70 | Positive | 28 | 5550 | 6h | 5 | corn silage | >50% |
| 315 | Podlaskie | 0.967 | Positive | 0.71 | Positive | 28 | 7600 | 12h | 5 | haylage | >50% |
| 316 | Podlaskie | 0.717 | Positive |  |  | 31 | 5500 | No grazing | 0 | corn silage | <50% |
| 317 | Podlaskie | 0.301 | Positive | 0.80 | Positive | 20 | 5300 | 12h | 5 | haylage | >50% |
| 318 | Podlaskie | 0.737 | Positive |  |  | 19 | 5500 | 24h | 5 | haylage | >50% |
| 319 | Podlaskie | 0.533 | Positive |  |  | 30 | 5500 | 12h | 5 | haylage | >50% |
| 320 | Podlaskie | 0.466 | Positive |  |  | 11 | 5000 | 12h | 5 | corn silage | <50% |
| 321 | Podlaskie | 0.738 | Positive | 0.92 | Positive | 17 | 5800 | 24h | 6 | haylage | >50% |
| 322 | Podlaskie | 0.127 | Negative |  |  | 34 | 7580 | 12h | 4 | haylage | <50% |
| 323 | Podlaskie | 0.611 | Positive | 0.68 | Positive | 34 | 6800 | 6h | 6 | haylage | >50% |
| 324 | Podlaskie | 0.509 | Positive |  |  | 40 | 7000 | 12h | 5 | haylage | <50% |
| 325 | Podlaskie | 0.882 | Positive | 0.98 | Positive | 23 | 4000 | 12h | 4 | haylage | <50% |
| 326 | Podlaskie | 0.970 | Positive | 0.64 | Positive | 32 | 7000 | 12h | 4 | haylage | >50% |
| 327 | Podlaskie | 0.784 | Positive |  |  | 40 | 6000 | 24h | 5 | haylage | >50% |
| 328 | Podlaskie | 0.725 | Positive |  |  | 10 | 6000 | 12h | 6 | haylage | >50% |
| 329 | Podlaskie | 0.762 | Positive | 0.66 | Positive | 40 | 6000 | No grazing | 0 | haylage | 0% |
| 330 | Podlaskie | 0.853 | Positive | 0.82 | Positive | 35 | 6500 | 12h | 5 | haylage | >50% |
| 331 | Podlaskie | 0.131 | Negative |  |  | 27 | 4500 | 12h | 6 | haylage | >50% |
| 332 | Podlaskie | 0.715 | Positive | 0.70 | Positive | 18 | 4000 | No grazing | 0 | corn silage | >50% |
| 333 | Podlaskie | 0.860 | Positive | 0.59 | Positive | 40 | 5000 | No grazing | 0 | haylage | 0% |
| 334 | Podlaskie | 0.366 | Positive | 0.51 | Positive | 35 | 3500 | No grazing | 0 | haylage | >50% |
| 335 | Podlaskie | 0.668 | Positive | 0.81 | Positive | 18 | 5800 | 12h | 5 | haylage | <50% |
| 336 | Podlaskie | 0.692 | Positive |  |  | 46 | 6000 | 6h | 5 | haylage | >50% |
| 337 | Podlaskie | 0.667 | Positive |  |  | 13 | 4000 | 12h | 5 | haylage | >50% |
| 338 | Podlaskie | 0.355 | Positive | 0.87 | Positive | 28 | 5500 | 12h | 5 | haylage | <50% |
| 339 | Podlaskie | 0.332 | Positive |  |  | 12 | 5000 | 24h | 6 | haylage | >50% |
| 340 | Podlaskie | 0.675 | Positive |  |  | 22 | 7000 | 12h | 5 | haylage | <50% |
| 341 | Podlaskie | 0.371 | Positive |  |  | 30 | 5200 | 12h | 5 | haylage | <50% |
| 342 | Podlaskie | 0.628 | Positive | 0.50 | Positive | 34 | 4600 | No grazing | 0 | corn silage | <50% |
| 343 | Podlaskie | 0.112 | Negative | 0.27 | Negative | 35 | 6500 | 12h | 5 | corn silage | <50% |
| 344 | Podlaskie | 0.331 | Positive | 0.11 | Negative | 16 | 4000 | No grazing | 0 | haylage | 0% |
| 345 | Podlaskie | 0.961 | Positive | 0.76 | Positive | 12 | 5200 | 6h | 4 | haylage | <50% |
| 346 | Podlaskie | 0.232 | Negative |  |  | 10 | 3500 | 24h | 5 | haylage | >50% |
| 347 | Podlaskie | 0.187 | Negative |  |  | 23 | 5300 | 24h | 5 | haylage | >50% |
| 348 | Podlaskie | 0.750 | Positive |  |  | 28 | 4500 | 24h | 5 | haylage | >50% |
| 349 | Podlaskie | 0.341 | Positive |  |  | 11 | 3000 | 12h | 5 | haylage | >50% |
| 350 | Podlaskie | 0.467 | Positive |  |  | 16 | 7000 | No grazing | 0 | corn silage | 0% |
| 351 | Podlaskie | 0.824 | Positive | 0.95 | Positive | 16 | 3700 | 24h | 6 | haylage | >50% |
| 352 | Podlaskie | 0.691 | Positive |  |  | 25 | 5500 | 24h | 5 | haylage | <50% |
| 353 | Podlaskie | 0.755 | Positive | 0.54 | Positive | 35 | 4000 | No grazing | 0 | haylage | 0% |
| 354 | Podlaskie | 0.752 | Positive | 0.47 | Negative | 21 | 6900 | 6h | 5 | haylage | <50% |
| 355 | Podlaskie | 0.785 | Positive | 0.88 | Positive | 21 | 5000 | 12h | 6 | corn silage | >50% |
| 356 | Podlaskie | 0.555 | Positive |  |  | 45 | 6800 | No grazing | 0 | corn silage | 0% |
| 357 | Podlaskie | 0.883 | Positive | 0.72 | Positive | 21 | 6000 | No grazing | 0 | haylage | 0% |
| 358 | Podlaskie | 0.714 | Positive | 0.68 | Positive | 95 | 8500 | No grazing | 0 | corn silage | >50% |
| 359 | Podlaskie | 0.730 | Positive |  |  | 30 | 7000 | 12h | 5 | haylage | >50% |
| 360 | Podlaskie | 0.671 | Positive | 0.01 | Negative | 18 | 5400 | 12h | 4 | haylage | >50% |
| 361 | Podlaskie | 0.592 | Positive |  |  | 39 | 6400 | 24h | 6 | haylage | <50% |
| 362 | Podlaskie | 0.706 | Positive |  |  | 26 | 5500 | 24h | 6 | haylage | >50% |
| 363 | Podlaskie | 0.333 | Positive | 0.43 | Negative | 82 | 8300 | No grazing | 0 | haylage | 0% |
| 364 | Podlaskie | 0.501 | Positive | 0.31 | Negative | 13 | 8500 | 12h | 4 | haylage | <50% |
| 365 | Podlaskie | 0.256 | Negative | 0.54 | Positive | 25 | 5000 | No grazing | 0 | haylage | >50% |
| 366 | Podlaskie | 0.601 | Positive |  |  | 17 | 4800 | 12h | 5 | haylage | <50% |
| 367 | Podlaskie | 0.667 | Positive |  |  | 13 | 3000 | 12h | 6 | haylage | >50% |
| 368 | Podlaskie | 0.416 | Positive |  |  | 14 | 4100 | 6h | 4 | haylage | >50% |
| 369 | Podlaskie | 0.418 | Positive |  |  | 18 | 5000 | 12h | 5 | haylage | <50% |
| 370 | Podlaskie | 0.694 | Positive |  |  | 41 | 4200 | No grazing | 0 | corn silage | 0% |
| 371 | Podlaskie | 0.596 | Positive |  |  | 36 | 4600 | 24h | 5 | haylage | <50% |
| 372 | Podlaskie | 0.901 | Positive | 0.87 | Positive | 10 | 3600 | 24h | 5 | corn silage | >50% |
| 373 | Podlaskie | 0.986 | Positive | 0.78 | Positive | 38 | 5500 | No grazing | 0 | haylage | 0% |
| 374 | Podlaskie | 0.119 | Negative | 0.33 | Negative | 17 | 5500 | No grazing | 0 | haylage | 0% |
| 375 | Podlaskie | 0.651 | Positive |  |  | 23 | 5200 | No grazing | 0 | corn silage | 0% |
| 376 | Podlaskie | 0.732 | Positive |  |  | 20 | 5000 | 12h | 4 | haylage | <50% |
| 377 | Podlaskie | 0.618 | Positive |  |  | 35 | 6000 | 6h | 3 | corn silage | <50% |
| 378 | Podlaskie | 0.660 | Positive |  |  | 19 | 5000 | No grazing | 0 | corn silage | <50% |
| 379 | Podlaskie | 1.051 | Positive | 0.81 | Positive | 38 | 6000 | 12h | 5 | haylage | <50% |
| 380 | Podlaskie | 0.267 | Negative |  |  | 25 | 6500 | 24h | 6 | haylage | <50% |
| 381 | Podlaskie | 0.611 | Positive |  |  | 32 | 6000 | No grazing | 0 | haylage | <50% |
| 382 | Podlaskie | 0.614 | Positive |  |  | 75 | 8500 | No grazing | 0 | corn silage | 0% |
| 383 | Podlaskie | 0.782 | Positive | 0.57 | Positive | 28 | 6000 | No grazing | 0 | haylage | >50% |
| 384 | Podlaskie | 0.348 | Positive |  |  | 25 | 4300 | 24h | 5 | haylage | >50% |
| 385 | Podlaskie | 0.308 | Positive |  |  | 18 | 7200 | 24h | 5 | haylage | >50% |
| 386 | Podlaskie | 0.714 | Positive |  |  | 46 | 8100 | 24h | 5 | haylage | >50% |
| 387 | Podlaskie | 0.226 | Negative |  |  | 28 | 7100 | 24h | 5 | haylage | >50% |
| 388 | Podlaskie | 0.568 | Positive |  |  | 15 | 4000 | 24h | 6 | haylage | >50% |
| 389 | Podlaskie | 0.710 | Positive | 0.35 | Negative | 11 | 6000 | No grazing | 0 | haylage | 0% |
| 390 | Podlaskie | 0.241 | Negative | 0.75 | Positive | 24 | 6200 | 24h | 4 | haylage | >50% |
| 391 | Podlaskie | 0.110 | Negative |  |  | 62 | 7300 | No grazing | 0 | haylage | 0% |
| 392 | Podlaskie | 0.879 | Positive | 0.86 | Positive | 32 | 7000 | 12h | 6 | corn silage | 0% |
| 393 | Podlaskie | 0.482 | Positive |  |  | 16 | 4000 | 12h | 5 | haylage | 0% |
| 394 | Podlaskie | 0.802 | Positive | 0.10 | Negative | 19 | 4200 | 12h | 5 | haylage | <50% |
| 395 | Podlaskie | 0.828 | Positive | 0.91 | Positive | 35 | 7250 | 12h | 5 | haylage | >50% |
| 396 | Podlaskie | 0.859 | Positive | 0.84 | Positive | 14 | 5500 | 12h | 5 | haylage | <50% |
| 397 | Podlaskie | 0.853 | Positive | 0.90 | Positive | 11 | 4200 | 12h | 5 | haylage | >50% |
| 398 | Podlaskie | 0.103 | Negative | 0.62 | Positive | 33 | 7800 | No grazing | 0 | corn silage | 0% |
| 399 | Podlaskie | 0.716 | Positive |  |  | 28 | 5500 | 12h | 5 | haylage | <50% |
| 400 | Podlaskie | 1.024 | Positive | 0.60 | Positive | 25 | 5000 | No grazing | 0 | haylage | 0% |
| 401 | Podlaskie | 0.411 | Positive |  |  | 60 | 8300 | 12h | 5 | haylage | 0% |
| 402 | Podlaskie | 0.781 | Positive | 0.66 | Positive | 30 | 4700 | 12h | 5 | haylage | >50% |
| 403 | Podlaskie | 0.558 | Positive |  |  | 18 | 5000 | 12h | 5 | haylage | <50% |
| 404 | Podlaskie | 0.712 | Positive |  |  | 30 | 3800 | 12h | 5 | haylage | >50% |
| 405 | Podlaskie | 0.639 | Positive | 0.67 | Positive | 14 | 6100 | 12h | 5 | haylage | >50% |
| 406 | Podlaskie | 0.506 | Positive |  |  | 15 | 4200 | 12h | 5 | haylage | >50% |
| 407 | Podlaskie | 0.906 | Positive | 0.97 | Positive | 32 | 6000 | 12h | 5 | haylage | <50% |
| 408 | Podlaskie | 0.871 | Positive | 0.68 | Positive | 26 | 3000 | 6h | 2 | haylage | 0% |
| 409 | Podlaskie | 0.114 | Negative | 0.39 | Negative | 12 | 8700 | No grazing | 0 | haylage | <50% |
| 410 | Podlaskie | 0.728 | Positive | 0.82 | Positive | 11 | 4000 | 12h | 6 | corn silage | >50% |
| 411 | Podlaskie | 1.013 | Positive | 0.78 | Positive | 14 | 6000 | 6h | 5 | haylage | <50% |
| 412 | Podlaskie | 0.646 | Positive |  |  | 28 | 6000 | 12h | 5 | haylage | >50% |
| 413 | Podlaskie | 0.178 | Negative | 0.61 | Positive | 49 | 5550 | No grazing | 0 | haylage | 0% |
| 414 | Podlaskie | 0.118 | Negative |  |  | 32 | 7000 | 6h | 6 | corn silage | <50% |
| 415 | Podlaskie | 0.360 | Positive |  |  | 40 | 5000 | 12h | 5 | haylage | <50% |
| 416 | Podlaskie | 0.377 | Positive |  |  | 29 | 5200 | 12h | 5 | haylage | <50% |
| 417 | Podlaskie | 0.719 | Positive |  |  | 16 | 4000 | No grazing | 0 | haylage | <50% |
| 418 | Podlaskie | 0.254 | Negative | 0.62 | Positive | 30 | 7200 | 12h | 3 | haylage | <50% |
| 419 | Podlaskie | 0.323 | Positive |  |  | 50 | 7100 | No grazing | 0 | haylage | 0% |
| 420 | Podlaskie | 0.299 | Positive |  |  | 15 | 6400 | 24h | 4 | haylage | >50% |
| 421 | Podlaskie | 0.640 | Positive | 0.91 | Positive | 17 | 1700 | 12h | 5 | haylage | >50% |
| 422 | Podlaskie | 0.677 | Positive |  |  | 15 | 2000 | 12h | 5 | corn silage | <50% |
| 423 | Podlaskie | 0.717 | Positive |  |  | 17 | 4500 | No grazing | 0 | corn silage | 0% |
| 424 | Podlaskie | 0.711 | Positive | 0.71 | Positive | 46 | 5200 | No grazing | 0 | corn silage | 0% |
| 425 | Podlaskie | 0.764 | Positive | 0.66 | Positive | 18 | 6600 | 12h | 4 | haylage | >50% |
| 426 | Podlaskie | 0.222 | Negative | 0.49 | Negative | 13 | 5000 | No grazing | 0 | haylage | <50% |
| 427 | Podlaskie | 0.743 | Positive | 0.69 | Positive | 21 | 6500 | No grazing | 0 | haylage | 0% |
| 428 | Podlaskie | 0.501 | Positive |  |  | 21 | 6000 | 24h | 5 | haylage | >50% |
| 429 | Podlaskie | 0.104 | Negative | 0.47 | Negative | 19 | 4500 | No grazing | 0 | haylage | 0% |
| 430 | Podlaskie | 1.011 | Positive | 0.79 | Positive | 23 | 5000 | 12h | 6 | haylage | >50% |
| 431 | Podlaskie | 0.851 | Positive | 0.58 | Positive | 28 | 6500 | No grazing | 0 | haylage | <50% |
| 432 | Podlaskie | 0.709 | Positive |  |  | 35 | 5500 | 6h | 5 | haylage | 0% |
| 433 | Podlaskie | 0.259 | Negative |  |  | 25 | 3500 | 6h | 4 | haylage | 0% |
| 434 | Podlaskie | 0.705 | Positive |  |  | 17 | 6000 | 6h | 3 | haylage | <50% |
| 435 | Podlaskie | 0.709 | Positive | 0.52 | Positive | 20 | 4500 | 12h | 6 | corn silage | <50% |
| 436 | Podlaskie | 0.719 | Positive |  |  | 20 | 4800 | 24h | 4 | haylage | >50% |
| 437 | Podlaskie | 0.709 | Positive |  |  | 30 | 6500 | 24h | 5 | haylage | >50% |
| 438 | Podlaskie | 0.684 | Positive |  |  | 24 | 5900 | 24h | 5 | haylage | >50% |
| 439 | Podlaskie | 0.852 | Positive | 0.97 | Positive | 13 | 4000 | 6h | 6 | hay | <50% |
| 440 | Podlaskie | 0.765 | Positive | 0.60 | Positive | 36 | 6100 | No grazing | 0 | haylage | >50% |
| 441 | Podlaskie | 0.917 | Positive | 0.83 | Positive | 18 | 3500 | No grazing | 0 | corn silage | 0% |
| 442 | Podlaskie | 1.011 | Positive | 0.71 | Positive | 39 | 5300 | No grazing | 0 | haylage | >50% |
| 443 | Podlaskie | 0.910 | Positive | 0.69 | Positive | 11 | 4500 | 12h | 5 | haylage | >50% |
| 444 | Podlaskie | 0.674 | Positive |  |  | 14 | 4700 | 24h | 5 | haylage | >50% |
| 445 | Podlaskie | 0.154 | Negative | 0.39 | Negative | 20 | 6200 | 12h | 6 | hay | >50% |
| 446 | Podlaskie | 0.805 | Positive |  |  | 21 | 5800 | 24h | 5 | haylage | >50% |
| 447 | Podlaskie | 0.611 | Positive | 0.01 | Negative | 23 | 2800 | No grazing | 0 | haylage | <50% |
| 448 | Podlaskie | 0.475 | Positive | 0.95 | Positive | 21 | 5100 | 12h | 6 | haylage | >50% |
| 449 | Podlaskie | 0.763 | Positive | 0.66 | Positive | 16 | 4800 | 12h | 5 | haylage | >50% |
| 450 | Podlaskie | 0.358 | Positive | 0.81 | Positive | 45 | 6500 | No grazing | 0 | haylage | 0% |
| 451 | Podlaskie | 0.103 | Negative |  |  | 25 | 6000 | No grazing | 0 | haylage | <50% |
| 452 | Podlaskie | 0.754 | Positive | 0.62 | Positive | 13 | 5000 | 12h | 6 | haylage | 0% |
| 453 | Podlaskie | 0.883 | Positive | 0.72 | Positive | 14 | 7500 | 12h | 2 | haylage | >50% |
| 454 | Podlaskie | 0.911 | Positive | 0.57 | Positive | 43 | 6200 | 12h | 5 | haylage | <50% |
| 455 | Podlaskie | 0.651 | Positive |  |  | 80 | 7000 | 6h | 4 | haylage | <50% |
| 456 | Podlaskie | 0.787 | Positive | 0.97 | Positive | 15 | 2500 | 12h | 6 | hay | >50% |
| 457 | Podlaskie | 0.415 | Positive | 0.54 | Positive | 26 | 5700 | 6h | 5 | haylage | <50% |
| 458 | Podlaskie | 0.783 | Positive | 0.81 | Positive | 21 | 6000 | 12h | 6 | haylage | >50% |
| 459 | Podlaskie | 0.703 | Positive |  |  | 25 | 5000 | No grazing | 0 | haylage | >50% |
| 460 | Podlaskie | 0.812 | Positive | 1.08 | Positive | 21 | 6000 | 12h | 5 | haylage | >50% |
| 461 | Podlaskie | 0.726 | Positive | 0.01 | Negative | 20 | 4000 | 12h | 1 | haylage | >50% |
| 462 | Podlaskie | 0.120 | Negative |  |  | 65 | 7000 | No grazing | 0 | haylage | 0% |
| 463 | Podlaskie | 0.451 | Positive |  |  | 13 | 3800 | 12h | 5 | corn silage | <50% |
| 464 | Podlaskie | 0.446 | Positive | 0.72 | Positive | 35 | 6500 | 12h | 5 | haylage | >50% |
| 465 | Podlaskie | 0.139 | Negative | 0.39 | Negative | 104 | 7000 | No grazing | 0 | corn silage | 0% |
| 466 | Podlaskie | 0.754 | Positive | 0.01 | Negative | 55 | 7000 | 6h | 5 | haylage | >50% |
| 467 | Podlaskie | 0.603 | Positive |  |  | 31 | 7000 | No grazing | 0 | haylage | 0% |
| 468 | Podlaskie | 0.152 | Negative | 0.36 | Negative | 109 | 8800 | No grazing | 0 | haylage | 0% |
| 469 | Podlaskie | 0.420 | Positive | 0.01 | Negative | 20 | 5500 | 12h | 5 | haylage | <50% |
| 470 | Podlaskie | 0.766 | Positive |  |  | 15 | 5900 | 12h | 4 | haylage | >50% |
| 471 | Podlaskie | 0.698 | Positive |  |  | 17 | 5000 | 24h | 2 | haylage | >50% |
| 472 | Podlaskie | 0.580 | Positive | 0.57 | Positive | 26 | 6300 | 12h | 5 | haylage | <50% |
| 473 | Podlaskie | 0.700 | Positive | 0.76 | Positive | 31 | 5700 | 12h | 5 | corn silage | >50% |
| 474 | Podlaskie | 0.614 | Positive | 0.36 | Negative | 29 | 6500 | 12h | 5 | corn silage | >50% |
| 475 | Podlaskie | 0.591 | Positive | 0.70 | Positive | 27 | 6000 | 12h | 5 | hay | >50% |
| 476 | Podlaskie | 0.823 | Positive | 0.66 | Positive | 15 | 4500 | 12h | 5 | haylage | <50% |
| 477 | Podlaskie | 0.588 | Positive |  |  | 10 | 5300 | 24h | 5 | haylage | >50% |
| 478 | Podlaskie | 0.753 | Positive | 0.57 | Positive | 31 | 7500 | No grazing | 0 | haylage | >50% |
| 479 | Podlaskie | 0.490 | Positive | 0.82 | Positive | 10 | 4500 | 6h | 5 | haylage | <50% |
| 480 | Podlaskie | 0.810 | Positive |  |  | 18 | 7000 | 12h | 5 | haylage | <50% |
| 481 | Podlaskie | 0.893 | Positive | 0.80 | Positive | 26 | 3000 | 12h | 6 | hay | >50% |
| 482 | Podlaskie | 0.755 | Positive | 0.73 | Positive | 15 | 6000 | 12h | 5 | haylage | <50% |
| 483 | Podlaskie | 0.645 | Positive |  |  | 15 | 5800 | 24h | 5 | haylage | >50% |
| 484 | Podlaskie | 0.618 | Positive |  |  | 12 | 6500 | 24h | 5 | haylage | >50% |
| 485 | Podlaskie | 0.679 | Positive |  |  | 55 | 5500 | 12h | 5 | corn silage | <50% |
| 486 | Podlaskie | 0.811 | Positive | 0.80 | Positive | 35 | 6700 | 6h | 5 | corn silage | <50% |
| 487 | Podlaskie | 0.399 | Positive |  |  | 16 | 5500 | 24h | 5 | haylage | >50% |
| 488 | Podlaskie | 0.834 | Positive | 0.84 | Positive | 39 | 5500 | 12h | 5 | haylage | <50% |
| 489 | Podlaskie | 0.312 | Positive |  |  | 25 | 6000 | 12h | 5 | haylage | <50% |
| 490 | Podlaskie | 0.295 | Positive | 0.49 | Negative | 29 | 6800 | No grazing | 0 | haylage | >50% |
| 491 | Podlaskie | 0.779 | Positive |  |  | 20 | 5550 | 12h | 5 | haylage | >50% |
| 492 | Podlaskie | 0.881 | Positive | 0.82 | Positive | 27 | 4500 | 12h | 4 | haylage | <50% |
| 493 | Podlaskie | 0.834 | Positive | 0.9 | Positive | 29 | 6500 | 12h | 6 | haylage | >50% |
| 494 | Podlaskie | 0.947 | Positive | 0.92 | Positive | 17 | 4100 | 12h | 6 | haylage | >50% |
| 495 | Podlaskie | 0.434 | Positive | 0.73 | Positive | 44 | 6550 | 12h | 5 | haylage | >50% |
| 496 | Podlaskie | 0.756 | Positive | 0.85 | Positive | 75 | 8100 | No grazing | 0 | haylage | 0% |
| 497 | Podlaskie | 0.330 | Positive | 0.07 | Negative | 25 | 6000 | No grazing | 0 | corn silage | 0% |
| 498 | Podlaskie | 0.822 | Positive |  |  | 12 | 4000 | 12h | 5 | haylage | >50% |
| 499 | Podlaskie | 0.775 | Positive | 0.71 | Positive | 28 | 5800 | 6h | 5 | haylage | <50% |
| 500 | Podlaskie | 0.360 | Positive | 0.66 | Positive | 32 | 5500 | 6h | 3 | corn silage | <50% |
| 501 | Podlaskie | 0.912 | Positive |  |  | 25 | 4245 | 24h | 5 | corn silage | >50% |
| 502 | Podlaskie | 0.742 | Positive | 0.95 | Positive | 17 | 4000 | 12h | 5 | haylage | >50% |
| 503 | Podlaskie | 0.783 | Positive | 0.60 | Positive | 25 | 6000 | No grazing | 0 | haylage | 0% |
| 504 | Podlaskie | 0.907 | Positive |  |  | 17 | 5000 | 12h | 6 | corn silage | <50% |
| 505 | Podlaskie | 0.404 | Positive | 0.74 | Positive | 42 | 6880 | 12h | 4 | haylage | >50% |
| 506 | Podlaskie | 0.580 | Positive |  |  | 52 | 8400 | 12h | 5 | haylage | >50% |
| 507 | Podlaskie | 0.265 | Negative |  |  | 12 | 5000 | 12h | 2 | corn silage | >50% |
| 508 | Podlaskie | 0.287 | Positive |  |  | 23 | 4200 | 12h | 5 | haylage | <50% |
| 509 | Podlaskie | 0.883 | Positive | 0.85 | Positive | 23 | 7000 | No grazing | 0 | corn silage | 0% |
| 510 | Podlaskie | 0.837 | Positive | 0.87 | Positive | 23 | 5000 | 12h | 6 | corn silage | >50% |
| 511 | Podlaskie | 0.838 | Positive | 0.84 | Positive | 40 | 5200 | 12h | 2 | haylage | >50% |
| 512 | Podlaskie | 0.583 | Positive |  |  | 13 | 3500 | 24h | 6 | haylage | >50% |
| 513 | Podlaskie | 0.108 | Negative | 0.15 | Negative | 32 | 6000 | No grazing | 0 | corn silage | 0% |
| 514 | Podlaskie | 0.166 | Negative |  |  | 12 | 4500 | No grazing | 0 | haylage | 0% |
| 515 | Podlaskie | 0.846 | Positive |  |  | 22 | 4000 | No grazing | 0 | haylage | <50% |
| 516 | Podlaskie | 0.854 | Positive | 0.72 | Positive | 40 | 5700 | 6h | 4 | haylage | >50% |
| 517 | Łódzkie | 0.095 | Negative | 0.41 | Negative | 32 | 9000 | No grazing | 0 | corn silage | 0% |
| 518 | Łódzkie | 0.143 | Negative | 0.28 | Negative | 18 | 7000 | No grazing | 0 | corn silage | 0% |
| 519 | Łódzkie | 0.103 | Negative | 0.06 | Negative | 60 | 8500 | No grazing | 0 | corn silage | 0% |
| 520 | Łódzkie | 0.291 | Positive | 0.88 | Positive | 11 | 5000 | 24h | 5 | corn silage | >50% |
| 521 | Łódzkie | 0.110 | Negative | 0.41 | Negative | 70 | 9200 | No grazing | 0 | corn silage | 0% |
| 522 | Łódzkie | 0.121 | Negative | 0.31 | Negative | 30 | 7000 | No grazing | 0 | corn silage | 0% |
| 523 | Łódzkie | 0.113 | Negative | 0.41 | Negative | 12 | 5000 | No grazing | 0 | corn silage | 0% |
| 524 | Łódzkie | 0.115 | Negative | 0.51 | Positive | 25 | 7500 | No grazing | 0 | corn silage | 0% |
| 525 | Łódzkie | 0.327 | Positive | 0.76 | Positive | 13 | 6000 | 24h | 4 | corn silage | <50% |
| 526 | Łódzkie | 0.132 | Negative | 0.31 | Negative | 18 | 7000 | No grazing | 0 | corn silage | <50% |
| 527 | Łódzkie | 0.436 | Positive | 0.79 | Positive | 16 | 6500 | 6h | 6 | haylage | <50% |
| 528 | Łódzkie | 0.122 | Negative | 0.62 | Positive | 60 | 8000 | No grazing | 0 | corn silage | 0% |
| 529 | Łódzkie | 0.080 | Negative | 0.39 | Negative | 20 | 5000 | No grazing | 0 | corn silage | 0% |
| 530 | Łódzkie | 0.162 | Negative | 0.63 | Positive | 12 | 7000 | No grazing | 0 | corn silage | 0% |
| 531 | Łódzkie | 0.201 | Negative | 0.91 | Positive | 10 | 5000 | 24h | 6 | haylage | >50% |
| 532 | Łódzkie | 0.473 | Positive | 0.84 | Positive | 17 | 6000 | 24h | 6 | corn silage | >50% |
| 533 | Łódzkie | 0.741 | Positive | 0.74 | Positive | 10 | 5550 | 24h | 6 | haylage | >50% |
| 534 | Łódzkie | 0.130 | Negative | 0.67 | Positive | 14 | 6000 | No grazing | 0 | corn silage | 0% |
| 535 | Łódzkie | 0.989 | Positive | 1.01 | Positive | 13 | 5000 | 6h | 5 | haylage | >50% |
| 536 | Łódzkie | 0.241 | Negative | 0.73 | Positive | 40 | 6000 | No grazing | 0 | corn silage | 0% |
| 537 | Łódzkie | 0.151 | Negative | 0.42 | Negative | 16 | 6000 | 24h | 6 | corn silage | <50% |
| 538 | Łódzkie | 0.618 | Positive | 0.92 | Positive | 10 | 6000 | 24h | 6 | corn silage | <50% |
| 539 | Łódzkie | 0.114 | Negative | 0.47 | Negative | 85 | 6800 | No grazing | 0 | corn silage | 0% |
| 540 | Łódzkie | 0.128 | Negative | 0.21 | Negative | 93 | 5550 | No grazing | 0 | corn silage | 0% |
| 541 | Łódzkie | 0.165 | Negative | 0.75 | Positive | 16 | 7500 | 24h | 6 | haylage | >50% |
| 542 | Łódzkie | 0.124 | Negative | 0.57 | Positive | 15 | 5000 | 6h | 5 | corn silage | >50% |
| 543 | Łódzkie | 0.394 | Positive | 0.30 | Negative | 14 | 5000 | 24h | 2 | haylage | >50% |
| 544 | Łódzkie | 0.171 | Negative | 0.71 | Positive | 33 | 9300 | 6h | 5 | corn silage | <50% |
| 545 | Łódzkie | 0.299 | Positive | 0.92 | Positive | 11 | 3000 | 24h | 6 | hay | >50% |
| 546 | Łódzkie | 0.185 | Negative | 0.71 | Positive | 34 | 7500 | No grazing | 0 | haylage | >50% |
| 547 | Łódzkie | 0.200 | Negative | 0.67 | Positive | 26 | 5500 | No grazing | 0 | corn silage | 0% |
| 548 | Łódzkie | 0.113 | Negative | 0.34 | Negative | 25 | 6500 | No grazing | 0 | haylage | 0% |
| 549 | Łódzkie | 0.119 | Negative | 0.52 | Positive | 19 | 5750 | No grazing | 0 | corn silage | 0% |
| 550 | Łódzkie | 0.275 | Positive | 0.79 | Positive | 50 | 6500 | 6h | 6 | corn silage | <50% |
| 551 | Łódzkie | 0.091 | Negative | 0.33 | Negative | 78 | 8400 | No grazing | 0 | corn silage | 0% |
| 552 | Łódzkie | 0.196 | Negative | 0.5 | Positive | 17 | 8370 | 6h | 3 | corn silage | <50% |
| 553 | Łódzkie | 0.145 | Negative | 0.30 | Negative | 23 | 9358 | No grazing | 0 | corn silage | 0% |
| 554 | Łódzkie | 0.528 | Positive | 0.61 | Positive | 11 | 5550 | 24h | 6 | hay | >50% |
| 555 | Łódzkie | 0.119 | Negative | 0.28 | Negative | 28 | 5550 | No grazing | 0 | corn silage | 0% |
| 556 | Łódzkie | 0.393 | Positive | 0.77 | Positive | 23 | 6500 | 6h | 5 | corn silage | <50% |
| 557 | Łódzkie | 0.245 | Negative | 0.75 | Positive | 20 | 5500 | 24h | 1 | corn silage | <50% |
| 558 | Łódzkie | 0.131 | Negative | 0.53 | Positive | 20 | 4000 | No grazing | 0 | corn silage | 0% |
| 559 | Łódzkie | 0.413 | Positive | 0.77 | Positive | 14 | 7000 | No grazing | 0 | corn silage | 0% |
| 560 | Łódzkie | 0.871 | Positive | 1.01 | Positive | 10 | 5000 | 24h | 6 | corn silage | >50% |
| 561 | Łódzkie | 0.416 | Positive | 1.02 | Positive | 10 | 6000 | 24h | 6 | hay | >50% |
| 562 | Łódzkie | 0.135 | Negative | 0.60 | Positive | 11 | 6000 | No grazing | 0 | haylage | 0% |
| 563 | Łódzkie | 0.328 | Positive | 0.74 | Positive | 10 | 3000 | 24h | 5 | haylage | >50% |
| 564 | Łódzkie | 0.618 | Positive | 0.64 | Positive | 11 | 6000 | No grazing | 0 | corn silage | <50% |
| 565 | Podlaskie | 0.478 | Positive | 0.72 | Positive | 10 | 4400 | 24h | 5 | hay | >50% |
| 566 | Podlaskie | 0.775 | Positive | 0.01 | Negative | 10 | 4200 | 12h | 1 | haylage | >50% |
| 567 | Podlaskie | 0.800 | Positive | 0.79 | Positive | 16 | 5800 | 24h | 5 | corn silage | >50% |
| 568 | Podlaskie | 0.258 | Negative | 0.49 | Negative | 25 | 4200 | 12h | 5 | corn silage | >50% |
| 569 | Podlaskie | 0.901 | Positive | 0.94 | Positive | 27 | 4800 | 24h | 5 | corn silage | >50% |
| 570 | Podlaskie | 0.713 | Positive | 0.71 | Positive | 10 | 4000 | 24h | 6 | haylage | >50% |
| 571 | Podlaskie | 0.590 | Positive | 0.79 | Positive | 11 | 4500 | 24h | 5 | hay | >50% |
| 572 | Podlaskie | 0.967 | Positive | 1.03 | Positive | 15 | 4860 | 24h | 5 | haylage | >50% |
| 573 | Podlaskie | 0.830 | Positive | 0.85 | Positive | 14 | 4800 | 12h | 5 | haylage | >50% |
| 574 | Podlaskie | 0.809 | Positive | 0.84 | Positive | 14 | 4960 | 24h | 5 | corn silage | >50% |
| 575 | Podlaskie | 0.608 | Positive | 0.75 | Positive | 18 | 5120 | 24h | 5 | hay | >50% |
| 576 | Podlaskie | 0.839 | Positive | 1.06 | Positive | 18 | 4600 | 24h | 5 | hay | >50% |
| 577 | Podlaskie | 0.540 | Positive | 0.91 | Positive | 10 | 5200 | 24h | 5 | haylage | >50% |
| 578 | Podlaskie | 0.870 | Positive | 0.64 | Positive | 32 | 3900 | 24h | 5 | haylage | >50% |
| 579 | Podlaskie | 0.479 | Positive | 0.90 | Positive | 24 | 6500 | 24h | 5 | haylage | >50% |
| 580 | Podlaskie | 0.993 | Positive | 0.91 | Positive | 10 | 4650 | 24h | 5 | haylage | >50% |
| 581 | Podlaskie | 0.801 | Positive | 0.46 | Negative | 25 | 4200 | 12h | 5 | haylage | >50% |
| 582 | Podlaskie | 0.618 | Positive | 0.76 | Positive | 32 | 6300 | No grazing | 0 | corn silage | <50% |
| 583 | Podlaskie | 0.650 | Positive | 0.79 | Positive | 10 | 5000 | 24h | 5 | haylage | >50% |
| 584 | Podlaskie | 0.466 | Positive | 0.83 | Positive | 10 | 4500 | 24h | 5 | haylage | >50% |
| 585 | Podlaskie | 0.941 | Positive | 0.88 | Positive | 12 | 3000 | 24h | 6 | haylage | >50% |
| 586 | Podlaskie | 1.516 | Positive | 0.75 | Positive | 25 | 5230 | 24h | 5 | haylage | >50% |
| 587 | Podlaskie | 0.801 | Positive | 0.83 | Positive | 17 | 5100 | 24h | 5 | haylage | >50% |
| 588 | Podlaskie | 0.916 | Positive | 0.79 | Positive | 22 | 4300 | 24h | 5 | haylage | >50% |
| 589 | Podlaskie | 0.767 | Positive | 0.91 | Positive | 14 | 5400 | 24h | 5 | hay | >50% |
| 590 | Podlaskie | 0.308 | Positive | 0.65 | Positive | 40 | 4400 | 24h | 5 | corn silage | >50% |
| 591 | Podlaskie | 0.078 | Negative | 0.97 | Positive | 32 | 4100 | 24h | 5 | haylage | >50% |
| 592 | Podlaskie | 0.069 | Negative | 0.85 | Positive | 15 | 4000 | 24h | 5 | haylage | >50% |
| 593 | Podlaskie | 0.830 | Positive | 1.00 | Positive | 32 | 4300 | 24h | 5 | haylage | >50% |
| 594 | Podlaskie | 0.983 | Positive | 1.00 | Positive | 17 | 4100 | 24h | 5 | hay | >50% |
| 595 | Podlaskie | 0.685 | Positive | 0.91 | Positive | 12 | 5550 | 24h | 5 | hay | >50% |
| 596 | Podlaskie | 0.722 | Positive | 0.92 | Positive | 16 | 4100 | 24h | 5 | haylage | >50% |
| 597 | Podlaskie | 0.723 | Positive | 0.89 | Positive | 30 | 5500 | 24h | 5 | haylage | <50% |
| 598 | Podlaskie | 0.983 | Positive | 0.01 | Negative | 24 | 4900 | No grazing | 0 | haylage | >50% |
