# Supplementary material for: Herd-level seroprevalence of Fasciola hepatica and Ostertagia ostertagi infection in dairy cattle population in the central and northeastern Poland
Source: BMC Vet Res. 2018 Apr 17;14:131. doi: 10.1186/s12917-018-1455-7 (PMC5905167; doi:10.1186/s12917-018-1455-7)
Supplement: Supplementary file 3 — Univariable analysis of herd-level risk factors for Fasciola hepatica exposure. Descriptive statistics and results of univariable statistical analyses comparing Fasciola hepatica bulk-tank milk (BTM) optical density ratio (ODR) between cattle herds with different characteristics. (DOCX 20 kb) [file 12917_2018_1455_MOESM3_ESM.docx]

Additional file 3. Univariable analysis of herd-level risk factors for *Fasciola hepatica* exposure

| Variable | n | *Fasciola hepatica* BTM ODR^a^ | p-value^b^ |
| --- | --- | --- | --- |
| Herd size | 598 | r_s_ = -0.02 | 0.625 |
| Grazing policy |  |  |  |
| No grazing | 264 | 0.177, 0.102-0.420 (0.002-1.024) |  |
| 6 hours a day | 66 | 0.416, 0.207-0.702 (0.090-1.013) | <0.001* |
| 12 hours a day | 179 | 0.621, 0.422-0.801 (0.078-1.051) | 0.006* |
| 24 hours a day | 89 | 0.608, 0.348-0.750 (0.069-1.516) | 0.999 |
| Length of grazing period (months) |  | r_s_ = 0.48 | <0.001 |
| Main roughage |  |  |  |
| Corn silage | 263 | 0.208, 0.112-0.475 (0.002-0.972) |  |
| Haylage | 311 | 0.580, 0.308-0.756 (0.060-1.516) | <0.001* |
| Hay | 24 | 0.690, 0.503-0.822 (0.043-0.983) | 0.485 |
| Proportion of grazing grass in diet |  |  |  |
| No grazing grass | 184 | 0.185, 0.104-0.420 (0.002-1.024) |  |
| Grazing grass <50% of all roughage | 223 | 0.404, 0.151-0.618 (0.026-1.051) | <0.001* |
| Grazing grass >50% of all roughage | 191 | 0.685, 0.416-0.801 (0.043-1.516) | <0.001* |
| Province |  |  |  |
| central (Łódzkie) | 204 | 0.146, 0.098-0.334 (0.002-0.989) |  |
| north-eastern (Podlaskie) | 394 | 0.596, 0.332-0.764 (0.062-1.516) | <0.001* |

^a^ given as the median, IQR and range in parentheses

^b^ in categorical variables applies to the comparison with the previous category

* explanatory variables included in the multivariable linear regression
