# Supplementary material for: Herd-level seroprevalence of Fasciola hepatica and Ostertagia ostertagi infection in dairy cattle population in the central and northeastern Poland
Source: BMC Vet Res. 2018 Apr 17;14:131. doi: 10.1186/s12917-018-1455-7 (PMC5905167; doi:10.1186/s12917-018-1455-7)
Supplement: Supplementary file 4 — Univariable analysis of herd-level risk factors for Ostertagia ostertagi exposure. Descriptive statistics and results of univariable statistical analyses comparing Ostertagia ostertagi bulk-tank milk (BTM) optical density ratio (ODR) between cattle herds with different characteristics. (DOCX 20 kb) [file 12917_2018_1455_MOESM4_ESM.docx]

Additional file 4. Univariable analysis of herd-level risk factors for *Ostertagia ostertagi* exposure

| Variable | n | Ostertagia ostertagi BTM ODR^a^ | p-value^b^ |
| --- | --- | --- | --- |
| Herd size | 324 | r_s_ = -0.15, | 0.006* |
| Grazing policy |  |  |  |
| No grazing | 134 | 0.465, 0.310-0.620 (0.010-0.880) |  |
| 6 hours a day | 33 | 0.680, 0.570-0.760 (0.010-1.010) | 0.001* |
| 12 hours a day | 106 | 0.770, 0.570-0.860 (0.010-1.080) | 0.723 |
| 24 hours a day | 51 | 0.840, 0.750-0.920 (0.300-1.060) | 0.012 |
| Length of grazing period (months) | 324 | r_s_ = 0.58 | <0.001* |
| Main roughage |  |  |  |
| Corn silage | 142 | 0.555, 0.360-0.730 (0.010-1.040) |  |
| Haylage | 159 | 0.710, 0.510-0.830 (0.010-1.080) | <0.001* |
| Hay | 23 | 0.830, 0.700-0.960 (0.390-1.060) | 0.010 |
| Proportion of grazing grass in diet |  |  |  |
| No grazing grass | 100 | 0.455, 0.300-0.640 (0.010-0.880) |  |
| Grazing grass <50% of all roughage | 98 | 0.615, 0.420-0.780 (0.010-1.040) | 0.002 |
| Grazing grass >50% of all roughage | 126 | 0.790, 0.670-0.900 (0.010-1.080) | <0.001* |
| Province |  |  |  |
| central (Łódzkie) | 136 | 0.505, 0.335-0.710 (0.010-1.040) |  |
| north-eastern (Podlaskie) | 188 | 0.725, 0.570-0.845 (0.010-1.080) | <0.001* |

^a^ given as the median, IQR and range in parentheses

^b^ in categorical variables applies to the comparison with the previous category

* explanatory variables included in the multivariable linear regression
